# British Society of Breast Radiology Annual Scientific Meeting 2018: Newcastle, UK. 04-06 November 2018

**DOI:** 10.1186/s13058-018-1052-5

**Published:** 2018-11-02

## Presentation Type: Oral

### OL.01 Digital breast tomosynthesis versus digital mammography (FFDM) in younger symptomatic women

#### Patsy Whelehan^1,2^, Kulsam Ali^2^, Sarah Vinnicombe^2,3^, Graham Ball^4,5^, Julie Cox^6^, Paul Farry^7^, Maggie Jenkin^8^, Dimitrios Kapsoulis^4,5^, Keith Lowry^9^, Stuart McIntosh^10,9^, Rachel Nutt^2,11^, Rachel Oeppen^12^, Michael Reilly^7^, Michaela Stahnke^12^, Jim Steel^8^, Yee Ting Sim^1^, Violet Warwick^2^, Louise Wilkinson^13^, Andrew Evans^1,2^

##### ^1^NHS Tayside, Dundee, United Kingdom; ^2^University of Dundee, Dundee, United Kingdom; ^3^Gloucestershire Hospitals NHS Foundation Trust, Cheltenham, United Kingdom; ^4^compandx, Nottingham, United Kingdom; ^5^Nottingham Trent University, Nottingham, United Kingdom; ^6^Sunderland Royal Hospital, Sunderland, United Kingdom; ^7^Altnagelvin Hospital, Londonderry, United Kingdom; ^8^Derriford Hospital, Plymouth, United Kingdom; ^9^Belfast City Hospital, Belfast, United Kingdom; ^10^Queen's University, Belfast, Belfast, United Kingdom; ^11^University of St Andrews, St Andrews, United Kingdom; ^12^Princess Anne Hospital, Southampton, United Kingdom; ^13^Oxford University Hospitals NHS Trust, Oxford, United Kingdom

###### **Correspondence:** Patsy Whelehan

**Background:** Young age and dense breasts are risk factors for false negative mammography even in the digital era, symptomatic settings included.

**Research question:** is digital breast tomosynthesis, alone or with FFDM, more sensitive than FFDM in women under 60 years with symptoms suggestive of breast cancer.

**Methods:** Women aged 25-59 referred for mammography in “one-stop” clinics, with clinical suspicion >20%, were prospectively consented into this UK multicentre study, designed to have 80% power to detect a 25% reduction in the false negative mammography rate. All participants had FFDM and DBT on Siemens Mammomat Inspiration units.

A retrospective multi-reader study using images from the 152 participants with cancer and 148 normal/benign participants was conducted. Two readers examined each case under each of three reading conditions: FFDM, DBT, DBT+FFDM. No reader read the same case twice; each read the same proportion of cases under each condition, in randomised order. Cancer sensitivity was calculated using a Monte Carlo simulation to extract subsets of cases to estimate the variance in sensitivity as well as calculate confidence intervals.

**Results:** Mean patient age was 48. Sensitivity for cancer was better for DBT than FFDM: DBT 90.7% (95% CI: 89.46-91.94), FFDM 87.55% (86.16-88.94), p=0.001. DBT+FFDM offered greater improvement over FFDM, at 91.3% (90.32-92.27), p<0.001. The difference between DBT and DBT+FFDM was not statistically significant (p=0.431).

**Conclusions:** DBT and DBT+FFDM are more sensitive than FFDM in younger symptomatic women. Statistical significance was strong but the clinical differences were small.

### OL.02 Opting into screening over the age of 70 years: Seeking evidence to support informed choice

#### Sarah Savaridas^1,2,3^, Patsy Whelehan^3,2,1^, Violet Warwick^2^, Andy Evans^2,1,4^

##### ^1^Ninewells Hospital, Dundee, United Kingdom; ^2^University of Dundee, Dundee, United Kingdom; ^3^East of Scotland Breast Screening, Dundee, United Kingdom; ^4^East of Scotland breast screening, Dundee, United Kingdom

###### **Correspondence:** Sarah Savaridas

**Introduction:** Breast cancer incidence increases with age. For women 50–70yrs, screening decreases breast cancer mortality. However, the risks and benefits of screening women >70yrs are unclear. This retrospective study seeks to provide evidence to help older women make informed choices.

**Methods:** The local breast cancer database was reviewed to establish cause of death in breast cancer patients over a 4-year period. Interrogation of the local breast-screening database was performed for women >70yrs over the same period.

**Results:** Across all ages, 1095 invasive cancers were diagnosed in the study period; 413 screen-detected, 679 symptomatic. There were 183 deaths (all causes); 82 in the screen-detected group, 101 in the symptomatic group; mean follow-up was 4.8yrs. Amongst women with symptomatic breast cancer the proportion of deaths due to breast cancer vs other causes significantly decreased with age (95.5% in <50yrs, 63.9% in 50-69yrs, 48.6% 70-79, 17.5% >80yrs; *p*<0.00001). In the study period, 5003 women aged >70yrs attended breast-screening, 184 (3.7%) were recalled to assessment, over half had an invasive procedure. Cancer detection rate was 13.5/1000 (25% DCIS).

**Conclusion:** In our service, most women >70yrs with symptomatic breast cancer die with, rather than of, breast cancer. The high proportion of other-cause deaths suggests limited scope for screening to extend life in this age-group. One in four screening cancers in women over 70 were DCIS, which, coupled with limited life expectancy, is likely to represent over-diagnosis in many cases. Data of this nature should be highlighted to women considering screening aged over 70.

### OL.03 Mucinous breast cancer: one disease or two?

#### Andrew Evans, Yee Ting Sim, Colin Purdie, Lee Jordan, Dawn Fleming

##### Dundee Medical School, Dundee, United Kingdom

###### **Correspondence:** Andrew Evans

**Purpose:** Interrogation of a large database of the shear wave elastography (SWE) features of over 2000 breast cancers showed mucinous cancer is the only tumour type which has a biphasic distribution of peri-tumoural stiffness on SWE. We hypothesised that the two groups may have different clinical, pathological and imaging features.

**Materials and methods:** 54 mucinous cancers were divided into soft and stiff groups using a cut off suggested by the stiffness distribution. The invasive size, presence of vascular invasion, nodal metastases, mode of presentation and age were documented. The mammographic and US features were assessed by two radiologists blinded the SWE findings. The pathology was reviewed to confirm the presence of pure mucinous cancer.

**Results:** Stiff mucinous cancers were significantly more likely than soft mucinous cancers to be associated with vascular invasion (18% vs 0%, p=0.01), nodal metastases (24% vs 4%, p=0.05) and large size (p=0.001). The frequency of a micropapillary pattern was similar in stiff and soft tumours. Age distribution and mode of presentation (screening or symptomatic) were also similar. Stiff tumours were less likely to be well defined on mammography (13% vs 34%, p=0.01) and less likely to have distal acoustic enhancement on US (0% vs. 10%, p=0.007).

**Conclusion:** The biphasic distribution of peri-tumoural stiffness splits mucinous breast cancer into two groups which have sufficiently different characteristics to suggest a clinically useful subdivision of mucinous cancers into those with and without an activated peri-tumoural stroma.

### OL.04 An analysis of 11.3 million screening tests examining the association between recall, biopsy and cancer detection rates in the English NHS breast cancer screening programme

#### Roger Blanks^1^, Matthew Wallis^2^, Jacquie Jenkins^3^, Rupert Alison^1^, Rosalind Given Wilson^4^

##### ^1^Cancer Epidemiology Unit, Nuffield Department of Population Health, Oxford, United Kingdom; ^2^Cambridge Breast Unit and NIHR Biomedical Research Centre, Cambridge, United Kingdom; ^3^Breast Screening Programme, Public Health England, London, United Kingdom; ^4^Dept of Radiology, St Georges University Hospital Foundation Trust, London, United Kingdom

###### **Correspondence:** Rosalind Given Wilson

**Purpose:** To model the association between cancer detection, recall and biopsy rates to understand the optimal balance of harm and benefit

**Materials and methods:**  Annual screening programme information for the 80 English breast screening units (11.3 million screening tests) supplemented by data from the Dutch screening programme. Linear and Non-linear regression models were used to examine and model the association between recall and needle biopsies per cancer detected.

**Results:** Higher recall rates are associated with higher needle biopsy rates and a rapidly increasing number of needle biopsies per invasive cancer detected. Our models suggest that at recall rates below 4.6% prevalent and 2.6% incident there is an increasing drop off in cancer detection rates.   At the other extreme for combined invasive/micro-invasive and high-grade DCIS detection rate reaches a near plateau above which the model predicts almost all recalls are false positive. However, for Low/intermediate grade DCIS (LIG) detection rate has no discernible plateau with detection rate increasing linearly at a rate of 0.12 (prevalent) and 0.18 (incident) per 1000 for every 1% increase in recall rate

Our model predicts that almost all invasive and high-risk DCIS will be detected with recall rates below 7% (needle biopsy rate 3%) at prevalent screen and below 4% (needle biopsy 1.5%) at incident screens.

**Conclusions:** Our model predicts that there is an optimum range for both recall and needle biopsy rates that optimises detection of life threatening cancers, whilst minimising harm.

### OL.05 The impact of conversion to digital mammography on cancer detection and recall rates in the English NHS breast cancer screening programme: analysis of 11.3 million screening tests

#### Roger Blanks^1^, Rosalind Given Wilson^2^, Rupert Alison^1^, Jacquie Jenkins^3^, Matthew Wallis^4^

##### ^1^Cancer Epidemiology Unit, Nuffield Department of Population Health, Oxford, United Kingdom; ^2^Dept of Radiology, St Georges University Hospital Foundation Trust, London, United Kingdom; ^3^Breast Screening Programme, Public Health England, London, United Kingdom; ^4^Cambridge Breast Unit and NIHR Biomedical Research Centre, Cambridge, United Kingdom

###### **Correspondence:** Matthew Wallis

**Introduction:** Full Field Digital mammography (FFDM) gradually replaced film screen mammography (FSM) in the English NHS breast cancer screening programme (NHSBSP) between 2008 and 2015. We report, for the first time, the impact of digital mammography in a large organised national screening programme without confounding by changes in other factors.

**Methods:** Annual screening data (KC62 returns) for each of the 80 units in the English NHSBSP over the seven years from 2009/10 to 2015/16 were used to examine the impact of changing from FSM to FFDM. Regression models were used to estimate percentage and absolute change in detection rates due to conversion from FSM to FFDM.

**Results:** The recall rate to assessment was almost unchanged by the introduction of FFDM. After conversion to FFDM the overall cancer detection rate rose by 14%, from 6.95 to 7.95 per 1000 screens, p<0.001. At prevalent (first) screens (women aged 45-52), FFDM increased the overall detection rate by 19% from 6.33 to 7.59 per 1000 (p<0.001) and for incident (subsequent) screens (women aged 53-70) by 13% from 7.11 to 8.02 per 1000. While there were increases in the detection of grade 1 & 2 invasive cancers, there was no change in the detection of grade 3 invasive cancers.

**Conclusion:** Digital mammography has increased the overall sensitivity of screening at the same recall rate as film screen and therefore improved the effectiveness of the screening process. However there has been no increase in detection of potentially life-threatening grade 3 cancers.

### OL.06 The relationship between breast screening readers’ real-life performance and their associated performance on the PERFORMS scheme

#### Yan Chen^1^, Leng Dong^1^, Eleanor Cornford^2^, Jacquie Jenkins^3^

##### ^1^Loughborough University, Loughborough, United Kingdom; ^2^Gloucester Breast Screening, Gloucester, United Kingdom; ^3^Public Health England, Sheffield, United Kingdom

###### **Correspondence:** Yan Chen

The performance of all breast screening personnel is recorded in the Breast Screening Information System (BSIS) which holds all national screening reader QA results for England. These individuals also take part annually in the PERFORMS self-assessment scheme where they report a series of challenging breast screening cases and their data are recorded and fed back to them to help their improve real life screening performance. Previously, a regional study showed strong correlation between real-life data and PERFORMS data for cancer detection rate. The relationship between these two sets of data were investigated nationally to determine how PERFORMS data relate to actual screening performance. Some 582 screeners consented to take part. Data were acquired from BSIS concerning their performance over a three-year period. Comparative data for each individual were also obtained from the PERFORMS database over the same time period and the relationship between the two sets of measures were examined. Some 533 participants’ data were successfully matched, validated and therefore included in this study. A Kendall's tau-b correlation was run to determine the relationship between the PPV values calculated from real-life data (cancer detected/ total recalls) over the past three years and the PERFORMS average PPV values over the same period. There was a strong, positive correlation between them, which was statistically significant (*τ*b = .141, *p* <.01) confirming that PERFORMS data accurately reflect real life screening performance. Therefore PERFORMS scheme can be used to give an early indication of any individual having difficulties and helped appropriately to improve.

## Presentation Type: Poster

### PA.01 Multiple papillomatosis of the breast: diagnostic and therapeutic dilemmas

#### Linda Metaxa, Charlotte Longman, Aisha Naseer, Georgios Exarchos, Jennifer Hu, Tamara Suaris

##### Breast Department, Bartshealth NHS Trust, London, United Kingdom

###### **Correspondence:** Aisha Naseer

Papilloma is the most common intraductal mass lesion of the breast and includes solitary intraductal papilloma, multiple papillomas (MP), papillomatosis, and juvenile papillomatosis. MP occur in approximately 10% of cases of intraductal papilloma. They can present in a number of ways including pain, lump and (serosanguineous) nipple discharge, or may be incidental. Imaging features vary and core biopsy and sometimes an additional vacuum biopsy is required, for definitive diagnosis.

MP compared to solitary lesions, tend to occur in younger patients, are less frequent, they are often bilateral, associated with nipple discharge and usually they are located at the periphery of the breast.

They carry an increased risk of malignancy and their management could be challenging. International Guidelines are not clear with regards to follow up or treatment. Some Breast societies, suggest annual follow up, as they are high-risk lesions, but it is not quite clear what imaging modalities (mammograms, US, MRI and combinations) should be used. Treatment options include both radiological and surgical excision of the lesions as well as surgical duct excision. However, when the lesions are innumerous, the only definitive treatment option is mastectomy but this option is considered aggressive and there is a controversy between doctors and centers.

The aim of our study is to present imaging features (mammograms, US, MRI) of multiple papillomatosis, and flag the diagnostic and therapeutic dilemmas, through a series of cases from our department and discuss scenarios of multiple papillomatosis, with or without atypia or DCIS in the prophylactic mastectomy.

### PA.02 Standardising core breast training across the North West of England

#### Sheetal Sharma, Alex Roberts

##### Royal Liverpool University Hospital, Liverpool, United Kingdom

###### **Correspondence:** Sheetal Sharma

There is a well recognised staffing crisis in breast radiology. Our experience is that providing good core training means that trainees are more likely to choose breast as a subspecialty. We distributed a survey to all trainees in the Mersey Training Programme to establish how much exposure they had had to breast radiology and at what stage. We also asked whether this had influenced their choice of subspecialty.

Following on from this we collaborated with breast radiologists from elsewhere in the North Western Deanery School of Radiology to draw up a comprehensive, practical guide to core breast training. This is divided into knowledge and skills and provides guidance on essential and desirable competencies.

Once approved at School board level this will be rolled out across the deanery and our hope is that it will ensure that all trainees in our region gain comprehensive, standardised breast radiology experience at ST2/3 level. This will allow them to make an informed decision regarding their future career options.

We will present the results of the survey as well as the main points from our core breast training document.

### PA.03 Modified Breast Ultrasound Phantom

#### Bernadette Bickley, Sarah Gascoigne

##### The Royal Wolverhampton NHS Trust, Wolverhampton, United Kingdom

###### **Correspondence:** Bernadette Bickley

Commercially available breast ultrasound phantoms are expensive and are known to degrade with repeated use. A homemade cheaper alternative is necessary in order to facilitate interventional training within a departmental setting. In the past, chicken breasts were used in order to replicate breast tissue, however, infection control concerns now prevent raw meat from being used for this purpose.

Jethwa et al have previously presented an informative poster with a recipe for a breast phantom and replication of the phantom was attempted using the recipe described. Technical difficulties arose and modifications to the original recipe were made to enable reproducible breast phantoms to be made.

### PA.04 Uncommon sites of breast cancer metastases

#### Reena Aggarwal^1,2^, Ayman Mahfouz^2^, Shama Puri^1^

##### ^1^Royal Derby Hospital, Derby, United Kingdom; ^2^University Hospitals of Leicester, Leicester, United Kingdom

###### **Correspondence:** Reena Aggarwal

**Introduction:** Breast cancer is the most common cancer in females, accounting for 31% of all cancers. The most common sites for breast cancer metastases are axial skeleton, lungs, liver and brain. It is important for radiologists to be aware of uncommon sites of breast cancer metastasis to allow correct diagnosis and appropriate patient management. We aim to present a pictorial review of the less common sites for breast cancer metastases from our institution.

**Method:** Patients with uncommon sites of metastatic disease were found from the breast cancer MDT. Imaging of all the patients was reviewed along with clinical history, tumour type and hormone receptor status. Histology was noted if the metastatic tumour site was biopsied. Response of metastatic disease to the treatment was also recorded.

**Results:** We include patients with metastasis to the orbit, pituitary infundibulum, leptomeninges, GI and GU tracts and calcaneum. We present these as case studies with a pictorial review.

**Conclusion:** With the rising incidence of breast cancer, it is important for radiologists to be aware of the uncommon sites of breast cancer metastases in their reporting, as accurate detection of metastases can affect patient management and prognosis.

### PA.05 Contrast Enhanced Digital Mammography: concepts, cases and highlights

#### Tharsi Sarvananthan, Emily Guilhem, Philippa Skippage, Fiona Hearn, Kirsten Stafford

##### Frimley Park Hospital, Surrey, United Kingdom

###### **Correspondence:** Tharsi Sarvananthan

**Teaching Points:**

1. Outline the principles and benefits of Contrast enhanced spectral mammography (CESM).
2. Illustrate multiple imaging cases where CESM has been a valuable diagnostic tool in our department.
3. Explore potential pitfalls of CESM and suggest methods of overcoming these issues.

**Outline:** Breast imaging techniques are rapidly evolving to supplement the increasing need for accurate assessment and direct appropriate treatment pathways. The currently utilised standard full-field digital mammography (FFDM) is a well-known cost-effective technique. However, dense breasts pose a diagnostic challenge. Breast MRI is currently regarded as the gold standard for breast cancer detection, staging and post-treatment monitoring. However, MRI is time consuming, costly, has limited routine availability, has several contraindications with potential lack of trained readers. CESM is an emerging tool that may be used as a faster and cheaper alternative, demonstrating promising early results. CESM combines contrast enhancement with digital mammography based on dual energy acquisitions to depict tumour vascularity with similar principles to MRI. Evidence from the literature has demonstrated increased sensitivity for cancer detection in comparison to FFDM and comparable sensitivity to MRI.

We present a series of cases demonstrating the use of CESM in various settings such as neoadjuvant assessment of tumour size, lobular breast cancer and as a problem solving tool in dense breasts. We also highlight the pitfalls of artefact posed by the presence of marker coils and case of reduced sensitivity for detecting additional sites of disease in multifocal cancer as has previously been described in the literature.

### PA.06 Mammary Fibromatosis: a retrospective review of cases presenting to our Breast Unit in the last 14 years

#### Anju Nandhra, Naveed Altaf, Kaushik Dasgupta, Anuradha Anand

##### North Tees Hospital, Stockton, United Kingdom

###### **Correspondence:** Anuradha Anand

**Background:** Breast Fibromatosis is a rare, benign, non-metastasizing, stromal tumour.

Aetiology is unclear with genetic and endocrine factors and trauma considered as possible causes. Treatment consists of wide local excision with adequate safety margins. Post-surgical recurrence rates upto 29% have been reported in some studies. Younger age, larger tumour size and positive margin status are factors associated with increased risk of recurrence.

**Methods:** We retrospectively reviewed our pathology database to identify cases. Hospital RIS/ PACS and pathology databases were reviewed to determine clinical presentation, imaging and pathological findings.

**Findings:** 10 cases of Breast Fibromatosis were identified; 8 females and 2 males.

Age at diagnosis ranged from 27 to 81 years, with a mean of 50.2 years.

8 cases presented with a symptomatic lump. 1 was recalled from screening. 1 had a mass noted on follow up mammograms after previous contralateral mastectomy for cancer.

On mammograms, 5 lesions were ill-defined and spiculate, mimicking malignancy. 2 lesions were not visible on mammogram. 3 patients did not have mammograms at diagnosis.

On Ultrasound, 4 cases demonstrated irregular hypoechoic masses mimicking malignancy, 2 demonstrated subtle distortion with surrounding hyperechogenicity and 1 demonstrated a well-defined lesion. 1 patient had a normal US. The remaining 2 did not have US.

All lesions were excised surgically. 1 patient is known to have had local recurrence so far.

**Conclusion:** Breast Fibromatosis often mimics malignancy on imaging. Though pathologically classified a B3 lesion, it requires wide local excision as it is locally aggressive with high local recurrence rates.

### PA.07 A pictorial review of Contrast Enhanced Spectral Mammography (CESM) cases and the potential impact of CESM on surgical decision making and patient outcome in the Belfast Trust

#### Elaine O'Boyle, Nicole Pierce, Elaine Davis, Louise Bamford

##### Belfast Trust, Belfast, United Kingdom

###### **Correspondence:** Elaine O'Boyle

**Learning Objectives:**

1. Review of CESM as a technique
2. Describe the salient imaging features on CESM cases from our institution
3. Highlight the clinical value of CESM appearances in aiding surgical decision making and potentially improving patient outcome.

**Background:** CESM is a novel breast imaging technique which combines an iodinated contrast agent with standard mammographic technique. As breast tumour growth is associated with angiogenesis CESM images the enhancement related to this neovascularity improving the lesion conspicuity and subsequently contributing to an unambiguous diagnosis.

Currently there are multiple indications for CESM in breast imaging with a role in both accurate diagnosis and as a problem solving tool; indications that had previously been reserved for breast MRI.

**Imaging Findings**:

We present a comprehensive series of CESM cases from the past four years in the Belfast Trust. We will depict the imaging features which aid accurate diagnosis and impact on patient management.

We will specifically illustrate cases which highlight the use of CESM for:

- Accurate assessment of disease extent and size in a newly diagnosed cases
- Problem solving- in patients with occult malignancies and in those with dense glandular breast tissue.

**Conclusion**: CESM is a growing technique for breast cancer detection. This pictorial review depicts the salient imaging features on CESM cases from our institution and highlights how it is an important adjunct investigation in breast cancer imaging, with the potential to impact both on surgical management and patient outcome.

### PA.08 Importance of mammographic interpretation to enable Band 6 & 7 Radiographers to deliver a high quality one stop service

#### Margaret Fletcher, Nisha Sharma, Anne Nielsen-Moody

##### Leeds Teaching Hospitals NHS Trust, Leeds, United Kingdom

###### **Correspondence:** Nisha Sharma

**Aim:** With limited financial resources, reduction in the availability of accredited courses and current staff shortages, we developed an in house mammographic module on image interpretation geared for Band 6&7 radiographers whose role involves delivering mammographic imaging as well as training and supporting junior members of the team.

**Method:**

A training package was developed comprising of:

- Training manual devised to including terminology and appearances.
- Modification of existing comments sheets in use within the department.
- Existing e learning package- this was deemed suitable as basic for the training.
- Reviewing previous imaging and reports to appreciate reporting styles.
- Sitting in cold reporting sessions with consultant radiographers.

How this was implemented: 2 x radiographers undertook the training over a 6 month period. Assessment on a test set of 20 cases. 6 months post assessment, trainees complete course evaluation questionnaire

For the assessment of 20 cases: appearances noted, free text report written and conclusion. The assessment was evaluated independently by 2 x consultant radiographers with consensus meeting for evaluation of results.

**Impact:** Both trainees had excellent results. This gave the trainees greater confidence in their work. They enjoyed the training and appreciated that they had been given the opportunity to do so.

They have a greater awareness of the importance of good technical quality imaging and engaged better with the reporting consultant radiographers/radiologists to ensure that mammographic abnormalities (real and compsoite) were appropriately worked up.

On completion certificates have been issued which can be used as evidence of CPD.

### PA.09 Incidental breast lesions on cross-sectional imaging: a pictorial review

#### Emma Hall, Sheetal Sharma

##### Royal Liverpool University Hospital, Liverpool, United Kingdom

###### **Correspondence:** Emma Hall

**Introduction:** The breasts are an important review area for every radiologist reporting cross-sectional imaging. With the ever-increasing demand for body imaging, there is an inevitable associated rise in the detection of incidental breast lesions. In this poster, we will present various cases of breast lesions detected on CT and MRI, as well as on Nuclear Medicine studies. The poster aims to illustrate imaging characteristics suggestive of malignancy to enable confident identification of breast lesions requiring further urgent investigation.

**Background:** Previously, any breast abnormality identified on cross-sectional imaging in our hospital was reported and referral to the symptomatic breast clinic was advised. In 2015, we implemented a code in our reporting system to highlight the case to the breast radiologists who would review the imaging, alongside any prior breast imaging. This has successfully reduced the number of patients requiring assessment in clinic and has provided interesting cases which will be presented alongside any subsequent dedicated breast imaging.

**Findings:** Breast lesions incidentally detected on non-breast imaging range from the benign to the malignant. Pertinent imaging features highly predictive of malignancy include irregular or spiculate margins and post-contrast enhancement. Lesions with features suggestive of benignity can be difficult to characterise on cross-sectional imaging and must be compared with any prior imaging to ensure stability.

**Conclusion:** Incidental breast lesions are increasingly identified on non-breast imaging. In many cases these are benign but we will present examples of malignant breast lesions and highlight the learning points associated with these cases.

### PA.10 Primary angiosarcoma or not?

#### Sighelgaita Rizzo, Nidhi Sibal, Rachel Howitt, Carol Ellen Holmes

##### Newcastle Breast Screening Unit, Royal Victoria Infirmary, Newcastle upon Tyne, United Kingdom

###### **Correspondence:** Sighelgaita Rizzo

Primary angiosarcoma of the breast is a very rare neoplasia, which unlike secondary angiosarcoma is not associated with previous radiation exposure. It accounts for less than 0.05% of all malignant tumours of the breast, however it is highly aggressive and carries a worse prognosis than mammary carcinomas.

Radiologically, angiosarcoma poses a significant challenge for diagnosis as there are no pathognomonic features seen on conventional imaging (ultrasound or mammography), and in fact findings can be very bland or even occult. Breast MRI is considered the most sensitive imaging modality. Pathology can also be non-specific, and therefore a close relationship between radiology and pathology is needed in order to optimise the diagnostic pathway of these patients.

Through the presentation of two cases, with similar histopathology, the difficulties in diagnosis with conventional imaging and pathology appearance will be discussed. Given the rarity and challenges of this condition, it is of high importance that suspected cases of primary angiosarcoma are appropriately referred to a specialist tertiary centre for optimal management.

A solid multi-disciplinary team approach is essential in the successful management of such cases.

### PA.11 Targeted axillary dissection using Iodine 125 seed localisation: the radiologists perspective

#### Carol Ellen Holmes^1^, Nidhi Sibal^2^

##### ^1^Newcastle Breast Screening Unit, Royal Victoria Infirmary, Newcastle upon Tyne, United Kingdom; ^2^Newcastle Breast Screening Unit, Royal Victoria Infirmaty, Newcastle Upon Tyne, United Kingdom

###### **Correspondence:** Carol Ellen Holmes

Targeted axillary node dissection is a technique used in larger centre’s internationally and is increasingly being used in centre’s in the UK. In post neo-adjuvant cases, it offers women less debilitating surgery and decreased morbidity.

Within our centre, Iodine-125 seed localisation is established as the standard localization technique for breast lesions. The ARSAC license has now been extended to allow seed localization of abnormal axillary nodes.

The aim of this poster is to outline how our procedures had to be adapted to include iodine-125 seed localisation of axillary nodes, including shoulder x-rays to demonstrate that the seed is in place. Post neo-adjuvant chemotherapy, the imaging appearances of the targeted node will have altered and identifying the marker clip may be difficult. Using examples, a discussion of the challenges faced in targeting nodes, in particular post neo-adjuvant chemotherapy will be included.

### PA.12 Psuedocirrhosis in metastatic breast cancer: an overview

#### Sophia Tincey, Dominic Yu, Anmol Malhotra

##### Royal Free Hospital NHS Trust, London, United Kingdom

###### **Correspondence:** Sophia Tincey

Pseudocirrhosis is an uncommon but significant complication observed in patients who have undergone systemic chemotherapy for breast carcinoma metastatic to the liver. Despite being a rare finding, it is a cause of morbidity in breast cancer patients and therefore it is important to improve awareness and understanding amongst breast radiologists to aid in appropriate and timely management.

Pseudocirrhosis is a process by which the liver progressively develops a morphological pattern that mimics classical cirrhosis and can cause significant difficulties in interpreting disease progression, stability and also response to chemotherapy. The appearances can be similar in all three scenarios on certain imaging modalities.

Radiologically it has similar appearances to macronodular cirrhosis, however pseudocirrhosis demonstrates a distinct pathophysiology. Pathogenesis is thought to be multifactorial although primarily related to the chemotherapy treatment of breast cancer. Multi modality techniques including ultrasound, CT, MRI and PET-CT will be used to demonstrate imaging characteristics, which include changes in hepatic contour, capsular retraction and complications from portal hypertension.

Working in a liver transplant unit we have had significant input into the imaging management of these patients from our hepatobilary radiology colleagues.

We will review these findings using radiological illustrations and discuss how to assess disease extent and progression with a guide to ongoing management.

### PB.02 Withdrawn

### PB.03 Withdrawn

### PB.04 A review of incidental breast lesions on CT, PET and MRI

#### Muhammad Mangat, Sarah McWilliams

##### St Georges Hospital, London, United Kingdom

###### **Correspondence:** Muhammad Mangat

We present a series of breast findings incidentally detected in patients undergoing cross sectional imaging for other pathologies. 30 patients presented to the symptomatic clinic for investigation of a lesion reported incidentally on predominantly CT or PET. Correlation with the cross- sectional image was essential for correct lesion correlation; evaluating the size of lesion, the nipple to lesion distance and ability to interpret the cross sectional image and plan where to target the ultrasound aided correct assessment.

In over 40 years old patients tomosynthesis helped locate the lesion and identified further lesions not seen on CT.

If biopsy was warranted, marker placement helped correlate with CT to enable correct lesion identification. Vivid enhancement on CT helped predict significant pathology. The study identified how breast malignancy can present in many remote sites and that recurrence can occur 30 years following treatment of the primary.

### PB.05 Assessing performance of MRI vacuum-assisted breast biopsy

#### Janice Yu Ji Lee, Fleur Kilburn-Toppin

##### Addenbrookes Hospital, Cambridge, United Kingdom

###### **Correspondence:** Janice Yu Ji Lee

Vacuum-assisted breast MRI-guided biopsy is required for indeterminate or suspicious breast lesions that cannot be identified on second look ultrasound, when this will affect patient management. We reviewed our tertiary referral centre’s performance of MRI biopsy with reference to the European Interdisciplinary Consensus Meeting following an initial audit from January 2013 to March 2016. Our standards were % of biopsy results discussed at MDT (100%) and % of B2 lesions undergoing further assessment - either short term MRI follow-up or re-biopsy (100%). We reviewed our electronic patient data system for MRI biopsies performed from April 2016 to May 2018. 66 biopsies were performed (69% increase from previous audit). 100% of results were discussed at MDT. 21/66 (32%) lesions were B5 at biopsy (23% in 2016). Of the remaining cases, 6 were B1, 27 B2, 11 B3 and 1 B4. All B3 and B4 lesions were surgically excised. Of the B1 lesions, 3 had follow up MRI, 2 underwent surgery and 1 agreed MDT decision to accept outcome due to existing medical co-morbidities. 13/27 B2 lesions (48%) underwent further assessment (20% in 2016). 7 had no documented follow up and 7 were lost to external follow up. Our results from our re-audit demonstrate an increased demand for MRI biopsy and cancer detection rate, in line with the literature. It also highlights the importance of BIRADS grading of MRI suspicion, allowing accurate assessment of concordance with biopsy results, and providing clear recommendation of follow up for non-concordant lesions and external referrals.

### PB.06 Radiographers’ agreement on mammography image quality parameters: experience of a Canadian breast screening program

#### Mohamed Abdolell^1,2^, Stephanie Schofield^2^, Ryan Duggan^1^, Kaitlyn Tsuruda^3^, Peter Brown^4,1^, Judy Caines^4^, Sian Iles^4,1^

##### ^1^Dalhousie University, Halifax, Canada; ^2^Nova Scotia Health Authority, Halifax, Canada; ^3^Cancer Registry of Norway, Oslo, Norway; ^4^IWK Health Centre, Halifax, Canada

###### **Correspondence:** Mohamed Abdolell

**Introduction:** Radiographers can vary substantially in their assessments of image quality. This study aims to evaluate the agreement between radiographers in evaluating image quality parameters to determine which parameters radiographers have more difficulty agreeing upon.

**Methods:** 1865 images were evaluated independently by 3 radiographers from a Canadian breast imaging centre. Pectoralis–nipple line (PNL) length (cm) was assessed, as well as the following binary quality parameters: inadequate inframammary fold (IMF), cut off, inadequate pectoralis, concave or thin pectoralis, other body parts, CC exaggeration, too high on IR, sagging, diagnostic quality, skin folds, missing posterior tissue, inadequate compression, motion, under exposure, inadequate sharpness, inadequate contrast, excess contrast, noise, and artefacts. Agreement was assessed using the Intraclass Correlation Coefficient (ICC) or weighted Kappa, as appropriate.

**Results:** Agreement between radiographers was highly variable from poor to almost perfect. PNL length exhibited almost perfect pairwise agreement (ICCs≥0.98) between radiographers. 3-way agreement between the radiographers was substantial to almost perfect for inadequate IMF, cut off, and inadequate pec (k=0.79, 0.74, and 0.64, respectively); moderate for concave or thin pectoralis, other body parts, CC exaggeration, and under exposure (k=0.59, 0.49, 0.43, and 0.43, respectively); and poor to fair for the remaining parameters. The range in pairwise agreement was narrow (<0.10) for most parameters, with a maximum of 0.67 for under exposure.

**Conclusion:** Radiographers demonstrate varying levels of agreement when evaluating image quality parameters. Agreement was better on parameters that were more objective.

### PB.07 LORIS the low risk DCIS trial: an update

#### Matthew Wallis^1^, On Behalf of TMG^2^

##### ^1^Cambridge Breast Unit and Cambridge NIHR Biomedical Research Centre, Cambridge, United Kingdom; ^2^CRCTU< University of Birmingham, Birmingham, United Kingdom

###### **Correspondence:** Matthew Wallis

A multi-centre, randomised (1:1), Phase III Trial of Surgery versus Active Monitoring for Low Risk Ductal Carcinoma in Situ (DCIS)

**Primary objective:** To assess whether active monitoring is non-inferior to Surgery, in terms of ipsilateral invasive breast cancer free survival time

**Primary outcome:** Ipsilateral invasive breast cancer free survival time

**Key Entry Criteria:**

1) Female, aged ≥ 46 years

2) Screen-detected or incidental micro calcification

3) Histologically confirmed diagnosis of non-high grade DCIS confirmed by local pathologist (for both breasts if bilateral disease)

By Small volume core biopsy and Vacuum Assisted Core Biopsy (VACB) **Or** Vacuum Assisted Core Biopsy (VACB) alone as first line diagnostic approach **Or** Small volume biopsy or VACB plus open diagnostic surgical biopsy (without clear margins) **Or** Open diagnostic surgical biopsy (without clear margins).

4) DCIS diagnosed ≤90 days before registration

5) Bilateral disease is permitted if both sides are confirmed to be non-high grade DCIS by local pathologist and the diagnosis is made within the same treatment episode

6) Able to give informed consent and comply with the trial schedule

7) Patient fit and willing to undergo surgery

8) Written Informed Consent obtained

We will update on current status of the trial and recruitment

### PB.08 Pre-NOSTRA: a feasibility study for the planned phase III No Surgery Trial (NOSTRA): an update

#### Daniel Rea^1^, Matthew Wallis^2^, On behalf of TMG^1^

##### ^1^CRCTU< University of Birmingham, Birmingham, United Kingdom; ^2^Cambridge Breast Unit and Cambridge NIHR Biomedical Research Centre, Cambridge, United Kingdom

###### **Correspondence:** Matthew Wallis

A large Phase III trial in which patients with multiple negative ultrasound guided tumour-bed biopsies after neo-adjuvant chemotherapy with combined multi-targeted anti HER2-directed therapy in ER-negative patients are randomised to surgery plus radiotherapy or radiotherapy alone is required.

The trial requires a Feasibility Study to explore both the willingness of patients to consent to randomisation and the accuracy with which multiple, post-treatment image-guided tumour-bed biopsies can detect residual disease.

1) To confirm concordance of local assessment of pathological complete response with central pathology review

2) To confirm that the multiple image-guided tumour-bed biopsies are able to exclude significant residual cancer burden

3) To explore the willingness of patients to be randomised

**Methods:** Eligible patients will receive neo-adjuvant chemotherapy with dual targeted anti-HER2 therapy. All patients will undergo protocol specified tumour-bed biopsies to systematically sample the tumour site. Samples will be locally reported and submitted for central review.

The willingness of those patients who have had a clinical response and negative post treatment image-guided tumour-bed biopsies to be randomised to either surgery and radiotherapy or radiotherapy alone will be explored via structured telephone interview prior to surgery. All patients in the feasibility study will undergo surgery.

The poster will provide an update of the trial status and describe the process for and documentation of tumour bed biopsies

### PB.09 The SMALL Trial: an update

#### Stuart McIntosh^1^, Matthew Wallis^2^, On Behalf of TMG^3^

##### ^1^Queen's University Belfast, Belfast, United Kingdom; ^2^Cambridge Breast Unit, Cambridge, United Kingdom; ^3^CRCTU< University of Birmingham, Birmingham, United Kingdom

###### **Correspondence:** Matthew Wallis

A Phase III, randomised, multi-centre trial addressing overtreatment of small, screen-detected breast cancer by comparing standard surgery with minimally invasive vacuum-assisted excision.

The SMALL trial aims to determine whether, in a selected low-risk population of women with small, biologically-favourable, screen-detected breast cancer:

1. The extent of surgical treatment can safely be reduced in the context of standard adjuvant radiotherapy and endocrine therapy.

2. The VAE technique is non-inferior in terms of requirement for a second operation to achieve complete resection of the cancer.

3. There is an acceptable local recurrence risk in the VAE arm with long-term follow up.

The study will also compare surgical complications, patient reported outcome measures and carry out a health economic evaluation of the procedures.

**Target population:**

Women > 50 years

Screen detected breast cancer < 15mm in size, ER/PR positive, HER2 negative

Patients randomised in a 2:1 ratio in favour of VAE

Single arm analysis of VAE patients to assess LR

**Sample size:** 801 patients to be recruited allowing for 5% drop out. In the randomised comparison of re-excision rates the probability of success in both arms is 80%: one-sided 2.5% alpha, 90% power to detect a maximum difference of 10%. 762 patients required. To exclude an undesirable local recurrence rate of 97% in the VAE cohort at 5 years with one-sided 2.5% alpha and 90% power requires 511 VAE patients to be followed for 3 years.

**Project timetable:** Duration: 9 years

**Recruitment:** 4 years with 18 month internal feasibility

### PB.10 Changing indications for breast MRI over a 6 year period in a district general hospital

#### Simon Lowes, Jane Potterton, Nikhil Birdi, Richard Morrell, Preet Hamilton, Alan Redman, Alice Leaver

##### Gateshead Health NHS Foundation Trust, Gateshead, United Kingdom

###### **Correspondence:** Simon Lowes

**Introduction**: In line with a general increase in workload in our breast unit, we have in particular noticed an increase in the number of breast MRI studies carried out. The aim of this study was to characterise the extent of this increasing demand over a 6 year period, together with any explanation for this.

**Methods**: Contemporaneous written records of completed breast MRI scans were analysed over the 6 year period covering 2012 to 2017 inclusive. Descriptive statistics were performed.

**Results**: A steady year on year increase was observed in the number of MRI scans carried out between 2012 and 2017, from 60 to 206. A major contributor to this trend was the increase in imaging for patients undergoing neoadjuvant chemotherapy (NAC), which increased from 2 to 56 scans. A smaller increasing trend was also seen for mammographically occult cancers, multifocal cancers, implant assessment, post cancer surveillance, and a variety of other miscellaneous reasons out with the standard NICE indications for breast MRI. The number of lobular cancers assessed peaked in 2015 but subsequently plateaued. There was no significant change in the number of scans carried out for size discrepancy on ultrasound versus mammography or for inflammatory cancers.

**Conclusion**: The 3.4-fold increase in MRI scans seen between 2012-2017 resulted from multiple factors, but notably by a large increase in scans for patients undergoing NAC. Although no plateau in MRI workload was seen over this period, preliminary data from the first half of 2018 suggests a plateau is now forming.

### PB.11 Male breast cancer in a UK breast unit: an audit of diagnostic and operative pathology in men over a 3 year period

#### Sally Athey, Simon Lowes, Preet Hamilton, Alan Redman, Alice Leaver

##### Gateshead Health NHS Foundation Trust, Gateshead, United Kingdom

###### **Correspondence:** Sally Athey

**Introduction:** Male breast cancers are rare, and over-investigation of benign breast conditions in males can cause harm and use valuable healthcare resources needed more urgently elsewhere.

We aimed to investigate our use of, and results of, breast histology in men, with a view to changing practice and improving patient care in our Unit.

**Methods:** All men that underwent breast biopsy or other breast histological analysis in our symptomatic breast service between Jan 2015-May 2018 were identified from electronic records. Clinical, imaging and pathology findings were reviewed. Descriptive statistics were performed.

**Results:** 423 men were imaged over the time period. 332 were aged >40 years (range 17-96).

28 men underwent diagnostic breast core biopsy, age range 24-96 years; 13 results were benign, 14 malignant, 1 indeterminate (confirmed as DCIS at surgery).

All men with diagnosed malignancy were >53 years (mean 71). All had bilateral mammogram and ipsilateral US performed at initial visit except one patient with a clinically fungating cancer. In all malignancies the imaging was classified indeterminate or suspicious (≥M3 and/or ≥U3), as was the clinical palpation score (≥P3).

There have been no subsequent breast cancer diagnoses in any males considered benign in this study.

**Conclusion:** Core biopsy is rarely performed on the male breast in our Unit, and has not yielded a malignant result under the age of 53. Male breast referral and imaging guidelines should take into account the very low risk of cancer and potential harm caused by over-investigation of the young male breast.

### PB.12 Comparison of preoperative ultrasound, mammographic, and MRI measurement of invasive lobular carcinoma with operative histology

#### Carpenter Sarah, Simon Lowes, Jane Potterton, Preet Hamilton, Alan Redman, Alice Leaver

##### Gateshead Health NHS Foundation Trust, Gateshead, United Kingdom

###### **Correspondence:** Jane Potterton

**Introduction:** Accurate pre-operative assessment of breast cancer is required to optimise treatment regimens. Lobular carcinoma is often infiltrative and therefore difficult to measure accurately.

This study aimed to determine the accuracy of imaging measurements in invasive lobular cancer patients who had undergone all 3 imaging modalities preoperatively.

**Methods:** MRI workstation records between January 2013 and December 2014 were used to identify all patients with invasive lobular carcinoma who had undergone MRI. Trust electronic imaging and pathology records were used to extract largest tumour dimensions for each imaging modality and final surgical pathology. Regression analysis and descriptive statistics were performed.

**Results:** Of 110 women diagnosed with invasive lobular carcinoma, 39 underwent preoperative ultrasound, mammogram and MRI; a further 3 were excluded as they were mammographically occult meaning no mammographic measurement was available.

Maximum tumour dimension on MRI had the highest correlation of the three imaging modalities when individually compared with final histology lobular carcinoma size (mammogram r^2^=43%, ultrasound r^2^=62%, MRI r^2^=68%).

Multivariate regression showed that the combination of ultrasound, mammogram and MRI was the most effective way of estimating size of malignancy in invasive lobular carcinoma (r^2^=70%, versus r^2^=61% for the combination of ultrasound and mammogram).

Ultrasound had a tendency to underestimate disease measurement, whereas MRI had a tendency to overestimate it.

**Conclusion:** The combination of ultrasound, mammogram and MRI most effectively predicts invasive lobular carcinoma largest dimension at final surgical pathology. Ultrasound tends to underestimate invasive disease size, as opposed to MRI where there is a tendency toward size overestimation.

### PB.13 Reactive sentinel lymph node enlargement post breast core needle biopsy in breast cancer patients undergoing MRI

#### Alice Leaver, Anju Nandhra, Alan Redman, Preet Hamilton, Simon Lowes

##### Gateshead Health NHS Foundation Trust, Gateshead, United Kingdom

###### **Correspondence:** Alice Leaver

**Introduction:** Ipsilateral axillary sentinel lymph node (SLN) histology is gold standard for staging breast cancer, guiding treatment. This study aims to explore whether anecdotal reactive enlargement of the SLN post-diagnostic core biopsy can be reliably identified on imaging (in this case MRI), and therefore possibly used to target the SLN.

**Methods:** All patients that underwent breast MRI following unilateral invasive lobular cancer diagnosis and normal axillary ultrasound from October 2012-June 2018 were identified from MRI workstation records. Other imaging and pathology identified from hospital electronic records. Neoadjuvant chemotherapy patients were excluded. MRI images were retrospectively reviewed, blinded to final surgical pathology, descriptive statistics performed.

**Results:** 174 eligible women underwent breast MRI between 2 and 51 days (mean 17, median 16) post diagnostic breast needle biopsy.

Only 22% (38/174) women had one (26), two (11) or more (1) ipsilateral prominent axillary lymph nodes and normal contralateral axilla on MRI (mean time to MRI 18 days, median 16 days; 8% (3/38) macrometastatic lymph node disease on final surgical pathology, versus 10% (17/174) of whole group studied).

In 66% (114/174) women axillary MRI was normal and symmetrical bilaterally, with prominent node(s) bilaterally in 9% (15/174). In 4% (7/174) the ipsilateral axilla appeared normal and the contralateral axilla demonstrated 1-2 prominent lymph nodes.

**Conclusion:** These findings do not suggest radiologically obvious and consistent reactive SLN enlargement post-breast biopsy within this population of patients and within our biopsy-to-MRI time frame, noting the limitations involved when comparing two different imaging modalities (ultrasound then MRI).

### PB.14 Review of ultrasound images in women with breast cancer between the ages of 30-36

#### Shazia Khan, Gemma Smith, Anne-Marie Wason

##### Bradford Teaching Hospitals Foundation NHS Trust, Bradford, United Kingdom

###### **Correspondence:** Shazia Khan

**Background:** In accordance to national standard practice, our department does not routinely biopsy masses in women under the age of 25 if Stavros criteria for a benign mass are fulfilled. In a previous audit, we found that extending the age limit of all women to aged 29 or under would not miss any cancer diagnoses. To take this forward, we looked to see if extending the age limit further to 36 years would have missed any cancers.

**Method:** All cases of breast cancer identified in women between the ages of 30 and 36 over 5 years were identified. Ultrasound images were identified and reviewed by 3 consultants against Stavros criteria to determine if a biopsy should be performed.

**Result:** 18 women were identified (age range 30-36) with all undergoing ultrasound at time of presentation. All cases identified a mass which was biopsied. Independent review of images confirmed that all the cancers biopsied in this group would not fulfil Stavros criteria. No patient had prior local imaging of the breast. No case was identified where the cancer was reported as benign with a subsequent cancer diagnosis.

**Conclusion:** Local experience supports the evidence based practice of not biopsying masses in women under the age of 25 which fulfil Stavros benign criteria. However, our findings suggest that extending this policy to the age of 29 would not have missed any breast cancers.

### PB.15 Accuracy of axillary lymph node ultrasound and core biopsy in patients with lobular carcinoma

#### Anju Nandhra, Simon Lowes, Preet Hamilton, Alan Redman, Alice Leaver

##### Gateshead Health NHS Foundation Trust, Gateshead, United Kingdom

###### **Correspondence:** Anju Nandhra

**Introduction:** Anecdotally, lymph node lobular carcinoma metastases are difficult to perceive at preoperative axillary lymph node ultrasound. We present an audit of lobular cancer patients, their preoperative staging of the axilla, operative axillary node histology, and any documented impact/change in patient management caused by a false negative axillary ultrasound.

**Method:** Retrospective analysis of multidisciplinary meeting records, hospital electronic pathology and imaging systems, were used to identify patients operated upon for invasive lobular carcinoma in our Trust from January 2012-March 2018. Neoadjuvant chemotherapy patients were excluded due to histological difficulty evaluating nodes post-chemotherapy. Descriptive statistics performed.

**Results:** 374 women were identified, of which 70 patients were excluded due to neoadjuvant chemotherapy.

All study patients underwent axillary ultrasound. The overall combined sensitivity and specificity of ultrasound/core biopsy in lobular cancer study patients was 63% and 100%, respectively.

163 patients were operated upon with a radiologically normal axilla; 91% (149/163) were lymph node negative at operative histology (sentinel lymph node biopsy), but 9% (14/163) did have lymph node macrometastases. In 64% (9/14) cases of unexpected lymph node metastasis, further axillary surgery was performed, and in 7% (1/14) axillary radiotherapy was planned. 29% (4/14) had no further axillary treatment.

**Discussion:** Lobular macrometatses can be present despite normal axillary ultrasound examination, and although this study demonstrates a nationally acceptable preoperative axillary staging sensitivity, unexpected macrometastases found at surgery do in many cases require further treatment.

### PB.16 Post-operative breast MRI: just how useful is it?

#### Nuala Healy, Ruchi Sinnatamby

##### Cambridge University Hospital Trust, Cambridge, United Kingdom

###### **Correspondence:** Ruchi Sinnatamby

**Purpose:** Breast MRI is increasingly requested to detect residual tumour in post-operative patients with positive margins. Post-operative interpretation, however, may be challenging. We reviewed breast MRIs performed post-operatively to determine our ability to detect additional disease distant from resection margins and evaluate whether it altered patient management.

**Methods and Materials:** A review of breast MRIs performed from August 2015 to June 2018 was undertaken to identify those performed post-operatively. Patients were referred following multidisciplinary review of conventional imaging and histology. Electronic records and imaging were reviewed to determine pathology and surgical outcomes.

**Results:** Of the 1257 breast MRIs during the study period, 33 were performed post-operatively, within 4 months of original surgery, in patients with positive margins who had not undergone pre-operative MRI imaging. 7 had already undergone initial margin re-excision prior to MRI. 7 cases (21%) revealed abnormal enhancement distant from the surgical cavity; 6 in the ipsilateral breast and in one, in the contralateral breast. 6 biopsies were performed following MRI. 5 were ipsilateral: 3 US and 2 MRI biopsies (1 benign, 1 LCIS, 2 high grade DCIS, and 1 IDC). In the contralateral case MRI biopsy revealed benign fibrocystic change. The seventh patient proceeded directly to mastectomy.

Following MRI, in 9 cases no further surgery was performed. 16 had re-excision, 1 of which subsequently underwent mastectomy. In total, 9 patients underwent mastectomy, four based on MRI findings.

**Conclusion:** Post-operative breast MRI can prove useful in detecting unsuspected distant disease and direct appropriate extent of further surgery.

### PB.17 Launch of the LIESL Trial – London Investigation into diElectric Scanning of Lesions. A review of early cases and lessons learnt from the set up and initiation of this trial using novel radiowave imaging technology (MARIA®)

#### Richard Sidebottom^1^, Donna Webb^2^, Briony Bishop^1^, Caroline Gillett^2^, Steve Allen^1^

##### ^1^The Royal Marsden Hospital, London, United Kingdom; ^2^Micrima Ltd, Bristol, United Kingdom

###### **Correspondence:** Richard Sidebottom

The Breast Unit Research Team at the Royal Marsden recently launched the LIESL trial which aims to determine the performance of the MARIA^®^ breast imaging system. There have been previous studies using MARIA^®^ with earlier iterations. In this trial we will gain experience in interpreting this novel breast imaging modality. Our experience and the data generated will also inform further development of the system by Micrima.

**Study set up and recruitment:**

The study has been designed to investigate the performance in 3 different cohorts.

Stream 1 Will determine the accuracy in identifying known breast cancers.

Stream 2 Will investigate the imaging characteristics of MARIA^®^ in patients attending the symptomatic clinic.

Stream 3 Will evaluate the utility of MARIA^®^ scans in assessment of patients undergoing neoadjuvant treatments.

The study will recruit 994 patients from women attending the breast imaging department, with 53 cases recruited to date.

**Image acquisition and interpretation:** We will discuss practicalities including scan time, optimum breast tissue position and patient acceptability.

Utilising an entirely new imaging modality is challenging and the team are continuing to develop understanding and experience in interpreting findings. Current viewing software requires three generated fields of view to be interpreted separately. Revised software will be available in July which allows faster and easier use. We will report on this also.

**Findings:** We will present a selection of cases from the trial so far in order to illustrate some of the strengths and challenges of this novel breast imaging technique.

### PB.18 Does baseline mammographic and peri-tumoural density influence the response of breast cancer to neoadjuvant chemotherapy (NACT)?

#### Andrew Evans, Violet Warwick, Yee Ting Sim, Colin Purdie, Celine Pourreyron, Caroline Michie, Sarah Vinnicombe

##### Dundee Medical School, Dundee, United Kingdom

###### **Correspondence:** Andrew Evans

**Introduction:** We aimed to assess the influence of baseline contralateral MD and the density of the local peri-tumoural tissues on the response of breast cancer to NACT.

**Methods:** Consecutive women with breast cancer treated with NACT who had a cancer free contralateral breast were the study group. Tumours were sub-classified according to immunohistochemistry as either triple negative, HER2 positive and ER positive HER2 negative.

Contralateral MD was retrospectively assessed using VOPLARA and the BIRADS classification and visual analogue scale (VAS) by breast radiologists blinded to treatment outcomes. The immediate peri-tumoural tissues were classified as either fatty, mixed or dense. Associations between MD and pCR rates were assessed using ROC curves.

48/240 tumours (20%) achieved a pCR. VAS and BIRADS assessed baseline MD did not show significant associations with the rate of pCR. Volpara assessed baseline density had a borderline association with the rate of pCR (AUC of 0.62, p=0.053).

VOLPARA assessed baseline MD for HER2 positive patients did show significant associations with the rate of pCR (AUC 0.70, p=0.004). Associations of VAS and pCR were borderline (AUC 0.60 (P=0.06). VAS, BIRADS and VOLPARA assessed baseline MD for the triple negative patients did not show significant associations with the rate of pCR. No asociation was found between the peritumoural density and response.

**Conclusion:** Women with HER2 positive breast cancer and very fatty breast have a very low rate of pCR compared to women with higher breast densities.

### PB.19 Role of staging in breast cancer patients receiving neoadjuvant chemotherapy (NACT)

#### Karen Lau, David Dodwell, Nisha Sharma

##### Leeds Teaching Hospital, Leeds, United Kingdom

###### **Correspondence:** Karen Lau

A retrospective audit of 388 patients that examines the role of staging (CT/bone scintigraphy or both) in patients receiving NACT. Of the 388 patients receiving NACT, 23% were Luminal A; 18% were Luminal B Her 2+ve, 21% were Luminal B Her 2-ve, 10% were HER 2+ve and 28% were triple negative. 1 case of unknown luminal subtype. 33% (128/388) had staging.

Within this group, 18% (23/128) had metastatic disease prior or during NACT. 18/23 cases were Luminal B and Triple negative cases. 3 cases were Her 2+ve, 1 Luminal A subtype and 1 in unknown luminal subtype. 22% (28/128) who had negative staging developed metastases after NACT.

67% (259/388) did not have any staging performed and 18% (47/259) developed metastases post treatment. 32/47 cases were from the Luminal B and Triple negative cases, of which 8/9 were under the age of 40.

98/388 women developed metastatic disease. 81/98 died from metastatic breast cancer, 2/98 died of other causes and 15/98 are living with metastases.

About 25% (98/388) of patients developed metastases after treatment.

Within the limitations of this study, there does not appear to be an appreciable difference between detection of metastases for those scanned prior or during NACT versus those who were scanned symptomatically after treatment, raising the question that staging is only necessary if patients are symptomatic in the context of NACT. Staging in asymptomatic women is of limited value for those receiving NACT.

### PB.20 Less is more. Can a small number of cores give an accurate result in the symptomatic setting?

#### Anwen Newland, Lubna Khalid, Sylvie Flais

##### London Northwest University Healthcare NHS Trust, London, United Kingdom

###### **Correspondence:** Anwen Newland

**Introduction:** There is no consensus regarding the minimal number of 14 gauge cores required at US guided biopsy. This is left to the discretion of the practitioner.

**Method:** Ultrasound guided biopsies performed at Ealing Hospital between January 2015 and June 2017 were reviewed. The number of cores obtained was recorded, and histology was reviewed, and compared with any subsequent histology. Any request from the reporting histologist for a repeat sample was recorded. An estimation of the workload for the histology department was obtained.

**Results:** 1 or 2 passes was performed in 82% of cases. 5 or more passes were performed in 1% of cases. Repeat biopsy was recommended by the MDT in 3 cases, and all were B2. (B3 biopsies requiring vacuum excision were not considered inadequate at initial sampling.) In our series, an increasing number of cores increased the likelihood of a B3 result, but not a B5 result.

**Discussion:** We consider a biopsy adequate once a sufficient amount of tissue is retrieved and the position of the needle is satisfactory on imaging. This is most often achieved with 1 or 2 passes. Our histologists do not have difficulty classifying lesions with a small number of cores. Increasing numbers of cores increases the histology reporting time and cost.

**Conclusion:** In our practice it appears safe and effective to limit the number of cores in a biopsy, saving time and money for our histology colleagues, and probably limiting the risk of biopsy complication.

### PB.21 Can the Stavros Criteria for benign breast masses be safely applied in women between 25 and 30 years?

#### Haleema Sadia, Nerys Forester

##### RVI, Newcastle, United Kingdom

###### **Correspondence:** Nerys Forester

**Introduction:** Breast masses in young women are usually benign and in under 25s biopsy can be safely avoided if Stavros criteria are met. We have explored whether Stavros criteria can be safely applied to 25-30-year-olds, to reduce the necessity for intervention.

**Methods:** Retrospective analysis of imaging of all ultrasound-guided core biopsies in 25-30-year-olds between 01/2012 and 12/2017. Captured images were used to determine whether Stavros criteria would apply. Pathological outcomes of core biopsies were correlated with radiological findings.

**Results:** Over 6 years, 194 patients underwent ultrasound-guided core biopsy (3 males). Lesion size ranged from 5-47mm. Images from 84/194 patients would have fulfilled the Stavros criteria. Biopsy results: 19 B1, 105 fibroadenomas and 57 miscellaneous B2 lesions (abscess, cysts, hamartomas, inflammation), 10 B3 lesions and 3 B5b. No breast cancers occurred in any lesions which would have fulfilled Stavros criteria in this cohort.

**Conclusion:** It is routine practice to use Stavros criteria to avoid biopsy in patients with benign masses under 25. This study demonstrates these criteria could be safely applied up to the age of 30. If used in this cohort, 84 patients could have been spared a biopsy without missing any cancers. However, 110 patients who did not satisfy the Stavros criteria would still have needed core biopsy to clarify the diagnosis, as usual practice, the majority of which were benign. As such, application of the Stavros criteria in 25-30-year-olds could safely reduce, but not eliminate, biopsy of benign breast masses.

### PB.22 Review of our initial use of tomosynthesis-guided biopsy - how did it help?

#### Dianne Lennox, Eman Hafez, Nerys Forester

##### RVI, Newcastle, United Kingdom

###### **Correspondence:** Nerys Forester

**Introduction:** As the use of digital breast tomosynthesis (DBT) increases, there is a need for biopsy methods to sample abnormalities only on DBT. We have used DBT since 2014, with DBT-biopsy since August 2016, in screening and symptomatic clinics. We have reviewed the use of DBT-biopsy to assess the management role of this new technique.

**Methods:** DBT-biopsies between 08/2016 and 05/2018 identified from PACS. Imaging findings, management decisions, and biopsy outcomes were reviewed.

**Results:** 61 patients underwent DBT-biopsy over 21 months (57 screening, 2 recalled from surveillance mammography, 2 incidental calcifications in symptomatic patients). 21 masses, 21 distortions, and 19 calcifications were biopsied. Reasons for using DBT-biopsy: 32 where the area was not identified on USS, 13 cases where DBT improved lesion accuracy and 16 for calcification where DBT was operator preference over stereotactic biopsy.

There were 16 B5 diagnoses (9 B5a, 7 B5b). In 8/16 cases, the mammographic lesion was not identified by ultrasound. In 2, DBT-biopsy allowed more accurate lesion identification (multiple lesions or initial ultrasound biopsy at inaccurate site). 6 cases (for calcification/clips) used DBT-biopsy at users discretion. In 45 cases, the DBT-biopsy was benign.

**Conclusion:** DBT-biopsy is a useful tool in the assessment of breast disease. It is particularly helpful in assessment of subtle distortions which were ultrasound occult, and where lesion localisation is difficult on conventional imaging. In addition, it provides a ‘belt and braces’ approach to low suspicion findings, where accurate benign biopsies can allow users to discharge the patient with increased confidence.

### PB.23 The SLOANE Project: an update

#### Matthew Wallis^1^, Karen Clements^2^, Janet Litherland^3^, Anthony Maxwell^4^, Nisha Sharma^5^, Alistair Thompson^6^

##### ^1^Cambridge Breast Unit and NIHR Biomedical Research Centre, Cambridge, United Kingdom; ^2^Screening Quality Assurance Service (Midlands & East), Public Health England, Birmingham, United Kingdom; ^3^West of Scotland Breast Screening Programme, Glasgow, United Kingdom; ^4^Nightingale Centre and Genesis Prevention Centre, Manchester, United Kingdom; ^5^Leeds Teaching Hospital NHS Trust, Leeds, United Kingdom; ^6^M.D. Anderson Cancer Center, Huston, TX, USA

###### **Correspondence:** Matthew Wallis

**Aims:**

- To improve knowledge about the diagnosis, treatment and clinical outcomes of screen detected carcinoma in situ and atypical hyperplasia
- Identify imaging and pathological features
- Identify (variations in) diagnostic and therapeutic practice
- To enable patients and health care professionals to make more informed choices regarding treatment in the future

Phase 1 of the Sloane Project (1 Apr 2003 and 31 Mar 2012) collected 13125 cases from 82 units (11331 with DCIS, 1135 with ‘atypia’, 659 await pathology form). The Sloane atypia audit from 1 Apr 2012 has collected 837 cases.

5 years follow up: Recurrence of DCIS or invasive breast cancer occurred in 6.8% (697/9,938), more commonly after BCS (7.8%) than mastectomy (4.5%; chi-square p<0.0010); 228 women (2.3%) developed contralateral DCIS or invasive breast cancer. (EJC 2018)

The length of recorded follow up in the main audit is now between 4 and 13 years and we will publish a series of paper incorporating these data.

The research arm is actively collecting tissue blocks for women who have developed an invasive recurrence and matched controls to identify biological markers of high risk disease.

There are other similar data sets but none have the level of information (particularly imaging) We need your help to make this data set world class. This year we will be contacting you for

- Clinical and imaging information on all subsequent events
- New cases of atypia
- Digital Images.

Sloane activity is ‘audit’ and we will be providing you certificates of participation for appraisal

### PB.24 Breast density analysis using DENSITAS software on interval cancers from the Welsh breast screening programme

#### Kate Gower Thomas^1^, Mohamed Abdolell^2^, Guy Stevens^1^, Dean McCarthy^1^, Kaitlyn Tsuruda^2^, Ryan Duggan^3^

##### ^1^Breast Test Wales, Cardiff, United Kingdom; ^2^University of Halifax, Nova Scotia, Canada; ^3^Densitas Inc, Halifax, Nova Scotia, Canada

###### **Correspondence:** Kate Gower Thomas

Breast density is well recognised as a risk factor for breast cancer with denser tissue more likely to develop cancer and less likely that such cancers are identified on mammography.

Breast densities on 277 consecutive interval cancers (ICA) screened at Breast Test Wales between 2012 and 2016 were analysed using DENSITAS 2.1.0 software, using processed images to calculate area density, percentage density, non-breast area and total breast area and BIRADS 5 density scores ABCD.

Classification and number of ICA were recorded; true 124 (44.7%), false negative 43 (15.5%), minimal signs 32 ( 11.5%), occult 20 (7%), unclassified 26 (9%) and 32 (11.6%) nonanalysed. Year one had 72 (26%) intervals, year two 129 (46.6%), year three 76 (27.4%); differing from NHSBSP standards when almost half of cases are year 3 following normal screen.

Of 268 cases with available information, there were 5 BIRADS A (1.7%), 83 BIRADS B (31%), 170 BIRADS C (63.3%) and 10 in BIRADS D (4%) categories.  Most ICA were observed in the third highest density group. The majority of true and false negative cases had breast densities of B and C grades.  In the highest density category, most (30%) were in the occult category.

Previous studies show a range of values in normal screened woman without cancer, where most were in the BIRADS B group (53%) and around 13% in BIRADS A category.  These data differ from ours and support the theory that higher breast density results in higher cancer incidence, and possibly higher interval cancer rates.

### PB.25 Does reviewing tomosynthesis images of breast resection specimens result in fewer positive margins at surgery?

#### Kate Gower Thomas, Michael Rees, Tara Davidson, Non Lawrence, Elizabeth Hubbard

##### Royal Glamorgan Hospital, Llantrisant, United Kingdom

###### **Correspondence:** Kate Gower Thomas

At our Centre, where >50% tumours are screen detected, 82% of patients undergo conservative surgical procedures. Second surgery for positive margins in breast cancer excision is required in nearly 20% of these, leading to delayed recovery, pressures on operating time and in patient stays and potential psychological stress for patients, knowing that 'they haven't got it all out.’

Using an Hologic Clarity HD system, all operative non-mastectomy breast cancer specimens localized due to impalpability, underwent mammography tomographic analysis between April and July 2018.  Images were reviewed at the time of surgery by the surgical team and/or radiologist and if the tumour was considered to be at margins, the surgeon would perform shaves from the tumour cavity, considered to be possibly margin positive.

60 consecutive specimens were identified, 5 excluded (localisation biopsies), 2 were incomplete. 53 were reviewed recording the mammographic feature, radiographic opinion of margin status; 79% good, 17% intermediate, 6% poor, surgical theatre notes and pathological reports.  17 cases (32%) underwent cavity shaves following review of the tomographic images; only one of which had positive pathological margin due to a coincidental, separate lesion. Of 37 without shaves, nine (24%) had positive margins and of these only one was considered tomographically close to margin.

Results were compared to conservative cases the previous year when 18% invasive cancers had second surgery for margins and 19% of DCIS only cases.

This early work may suggest that tomographic review of specimens might contribute to reducing second surgery, although further work is required.

### PB.26 A safe and efficient way to save time and money

#### Sylvie Flais^1^, Anwen Newland^2^, Shyamala Fernandez^1^, Lubna Khalid^1^

##### ^1^Ealing Hospital, London North West University Healthcare NHS Trust, Southall, United Kingdom; ^2^Ealing Hospital, London North West University Healthcare NHS Trust, Southall, United Kingdom

###### **Correspondence:** Sylvie Flais

**Introduction:** Patients younger than 25 with a solid mass that fulfils benign criteria (BI RADS 3, graded U2 in our institution) do not require a biopsy. Following the introduction of shear wave elastography (SWE), it has been suggested that patients younger than 40 could also avoid a biopsy providing grey scale and SWE are benign and concordant. How much time and money could this save?

**Material and method:** All U2 masses biopsied between January 2015 and June 2017 were audited. Patients’ age, imaging grade, number of passes and histology were collected. The average time and cost required in imaging and histology were estimated.

**Results:** 283 biopsied U2 lesions were included. 166 patients were younger than 40 (58%). 161/166 (98%) had a B2 histology. 2 patients had a B1 result (normal tissue, lipoma), 3 had a B3 result (papilloma for 2, ADH upgraded to low grade DCIS after vacuum excision for 1).

The costs of disposable material in our unit is £22 per biopsy and £27 for histology technical preparation. Imaging biosy time is 10 min and histology time is 5 min for a fibroadenoma and 2 cores of tissue (estimated average). SWE takes 2 minutes to perform.

**Discussion:** If 80% of biopsies had been avoided, this would have saved a minimum of £6500 and 29 hours of doctors’ time in 29 months. Not sampling U2/BI RADS 3 lesions in all breasts units in the UK would save a significant amount of time and money.

### PB.27 When is it safe for the multi-disciplinary team to accept a fibrosis result following symptomatic assessment?

#### Sylvie Flais, Anwen Newland, Shyamala Fernandez, Grigorios Mitsopoulos

##### Ealing Hospital, London North West University Healthcare NHS Trust, Southall, United Kingdom

###### **Correspondence:** Sylvie Flais

**Purpose:** According to Royal College of Pathology and NHS Breast Screening guidelines a B1 biopsy result is acceptable by the multi-disciplinary team (MDT) if concordant with the sampled lesion. In the symptomatic setting, can fibrosis be accepted as concordant and should it be graded B1 or B2?

**Method:** Fibrosis/hyalinisation without other features obtained following an US guided biopsy at Ealing Hospital between January 2015 and June 2017 were audited. The clinical and imaging grades and the histology result were recorded. If the MDT decided to repeat the biopsy, the technique and result of the repeat biopsy was included.

All imaging and histology were reviewed to document the evidence of adequate sampling as well to confirm the grade given initially.

**Results:** 755 biopsies were performed. In 42 cases fibrosis or hyalinisation were the only histology features, graded B2 in 41/42 (98%). US grading was 2-3 in 39/42 (93%), 4 in 3/42. The MDT advised a repeat biopsy in 3 cases: 1 diabetic mastopathy at repeat, 1 fibrosis, 1 could not be performed as the lesion was not seen. 1 patient had follow-up imaging for 2 years, unchanged.

**Discussion:** Fibrosis can be graded B1 or B2. We discuss the reasons for a B2 grade. In our institution the MDT decision of accepting fibrosis as concordant appears to be influenced by the histology grade as well as the most likely benign imaging features.

**Conclusion:** A fibrosis result is acceptable when the lesion has been adequately sampled and imaging features are consistent.

### PB.28 Does breast implant MRI add any value to ultrasound assessment?

#### Nikhil Birdi, Preet Hamilton, Alice Leaver, Simon Lowes, Alan Redman

##### Gateshead Health NHS Foundation Trust, Gateshead, United Kingdom

###### **Correspondence:** Alan Redman

**Introduction:** The RCR Guidance on screening and symptomatic breast imaging (June 2013) states, “MRI is the modality of choice to assess the integrity of breast implants”. The aim of this study is to see whether breast MRI adds value to ultrasound assessment of implant integrity.

**Methods:** All patients who had undergone ultrasound followed by MRI to assess integrity of single lumen breast implants between January 2012 and May 2018 identified retrospectively from breast clinic records. Imaging reports reviewed on hospital electronic patient information systems. Descriptive statistics performed.

**Results:**

93 implants were identified in 62 females.

37 implants were reported as intact on ultrasound; MRI agreed in 100%.

25 implants were reported as cannot rule out intracapsular (IC) rupture on ultrasound; 23 (92%) were intact on MRI, and 2 (8%) were reported as uncertain for IC rupture on MRI.

16 implants were reported as suspicious for IC rupture on ultrasound; on MRI, 14 (88%) were reported as definite IC rupture, 1 (6%) was reported as suspicious and 1 (6%) was reported as uncertain for IC rupture.

10 implants reported as definite IC rupture on ultrasound; all (100%) confirmed on MRI.

2 implants reported as suspicious for extracapsular (EC) rupture on ultrasound. 1 reported as definite IC, but not EC, rupture on MRI. 1 reported as suspicious of EC rupture on MRI.

3 implants reported as definite EC silicone on ultrasound, confirmed in all 3 on MRI.

**Conclusion:** In our unit, MRI adds little to ultrasound in the assessment of implant integrity.

### PB.29 Fat necrosis: a review of imaging and pathology findings in a UK breast unit

#### Sue Tan, Sally Athey, Alice Leaver, Simon Lowes, Preet Hamilton, Alan Redman

##### Gateshead Health NHS Foundation Trust, Gateshead, United Kingdom

###### **Correspondence:** Alice Leaver

**Introduction:** Fat necrosis of the breast is a benign condition, but imaging findings can sometimes be variable and indeterminate. In our Trust, where biopsy is considered unnecessary on imaging, follow up imaging is advised for confirmation.

We present a retrospective audit of our management of patients with likely fat necrosis on imaging.

**Methods:** All patients with fat necrosis reported on first imaging between January 2015 and December 2016 were identified retrospectively from breast clinic records. Imaging and pathology findings reviewed on hospital electronic patient information systems. Descriptive statistics performed.

**Results:**

Ninety-six patients (89 female, 7 male) were identified from breast clinic records.

31% (30/96) patients had one imaging attendance, 55% (53/96) had two, and 14% (13/96) >2 attendances.

43% (13/30) underwent biopsy at their first imaging attendance, with two malignancies detected.

69% (66/96) patients were assigned to follow up imaging, at which 74% (49/66) demonstrated radiological improvement or complete resolution. 26% (17/66) underwent biopsy at second visit, 8% (5/66) at a later visit. One follow up biopsy result (5%, 1/22) was malignant, all others benign (95%, 21/22). On imaging review, the malignant follow up biopsy was delayed due to image misinterpretation.

There has been no further cancer identified in this cohort of patients to date (June 2018).

**Conclusion:** Core biopsy should be performed at first imaging attendance if clinico-radiological findings are not entirely consistent with fat necrosis.

Our findings do not support routine follow up imaging of fat necrosis patients who do not meet imaging criteria for biopsy.

### PB.30 Blurred Mammograms: investigating technical recall decisions through a multi-reader study

#### Patsy Whelehan^1,2^, Sarah Vinnicombe^3,1^, Violet Warwick^1^, Maria Pampaka^4^, Katrin Brauer^2^, Jill Mitchell^2^, Yee Ting Sim^2^, Chris Tromans^5^, Melissa Hill^6^, Andy Evans^2,1^

##### ^1^University of Dundee, Dundee, United Kingdom; ^2^NHS Tayside, Dundee, United Kingdom; ^3^Gloucestershire Hospitals NHS Foundation Trust, Cheltenham, United Kingdom; ^4^University of Manchester, Manchester, United Kingdom; ^5^Volpara Health Technologies Ltd., Oxford, United Kingdom; ^6^Volpara Health Technologies Ltd., Issy les Moulineaux, France

###### **Correspondence:** Patsy Whelehan

**Introduction:** Motion blur is a problem in mammographic screening. Blur accepted or overlooked at reading may risk lesions being missed.

**Aim:** To understand reader decision-making on technical recall (TR) of blurred images.

**Methods:** Twenty-five 4-view mammograms containing blurred images were selected by an experienced breast radiologist. Five additional screen readers each reviewed all 100 anonymised images.

Readers graded images as “not blurred”, “blurred/TR”, or “blurred/no TR”, scored blur severity (0-100 scale), indicated blurred areas, and graded BI-RADS® density. Volumetric breast density was computed (Volpara, v1.5.2).

Reader agreement was analysed by intraclass correlation coefficient (ICC) and Fleiss’ kappa; multiple logistic regression was used to identify best predictors of TR in the blurred images.

**Results:** Images were classified as blurred in 141/500 reads (38/100 images). TR was required in 65/141 reads (22/100 images). In the images where at least one reader reported blur, severity score ICC was 0.62 (95% CI: 0.47-0.76); Fleiss kappa for TR agreement: 0.46. Mean severity score was 20 in non-TR and 37 in TR.

Severity best predicted TR (χ^2^=116.9, p<0.001, Pseudo R^2^=0.61) and dominated the regression model. Excluding severity from the model revealed significant effects of reader, size of blurred area and patient age, when also accounting for location of blur, and Volpara and BI-RADS® density (Pseudo R^2^= 0.43).

**Conclusion:** The dominant factor predicting TR for blur was readers’ assessment of severity. Size of the affected area and breast density were predictive only with severity excluded from the analysis. Reader agreement was moderate.

### PB.31 Early results of the new stereotactic guided breast biopsy Brevera system- a breakthrough in micro-calcification sampling efficiency?

#### Tharsi Sarvananthan, Ronit Uzvolk, Rosalind Felton, Mia Morgan, William Teh

##### North London Breast Screening Services, London, United Kingdom

###### **Correspondence:** Tharsi Sarvananthan

**Background:** The Brevera system aims to streamline breast biopsy using real-time verification of micro-calcification (MCC). This allows early and confident confirmation of representative MCC potentially minimising the number of samples required, hence reducing procedure time, patient discomfort and complications. We review whether real-time imaging allows early cessation of sampling once MCC is identified rather than acquiring the usual 12 samples without affecting biopsy accuracy.

**Method:** The Brevera system was introduced to the North London Breast Screening in January 2018. All women underwent a minimum of 12 samples or more with confirmation of calcification. Cassettes numbered in chronological order with real-time confirmation of MCC using Brevera was compared to the corresponding histology.

**Results:** Seventy-four MCC clusters were sampled using the Brevera system between January-July. 95% of these were classified as indeterminate/R3 with 4% as R4 and 1% as R5. The average cluster size was 12mm. The majority were B2 benign (56%, n=42), followed by B5 (26% n=19) and B3 (15% n=11). Of the malignant lesions, 63% (12/19) had concordant histology with real-time first presence of MCC using Brevera. However, 21% (4/19) had definitive histology on later cassettes despite earlier presence of MCC’s. Conversely, 3/19 samples had definitive histology within earlier cassettes than identified by the Brevera system.

**Conclusion:** Although the Brevera system allows real-time verification of representative calcification, at least 21% of cancers would have been underdiagnosed as non-malignant if sampling was stopped earlier. Our early results support obtaining the standard 12 samples in order to obtain accurate final histology.

### PB.32 Lessons learnt from same site cancer over a 5-year screening period - a pictorial review

#### Tharsi Sarvananthan, William Teh

##### North London Breast Screening Services, London, United Kingdom

###### **Correspondence:** Tharsi Sarvananthan

**Background:** Same site cancers or false negative assessments (FNA) can occur despite sufficient assessment and present as interval or screen detected cancer at the subsequent screening round. In minority of patients, cancers are not diagnosed at the initial assessment due to misinterpretation or inadequate assessment. We present a pictorial review with learning points from unsatisfactory assessments of same site assessments (SSA) over 5-year period at North London Breast Screening.

**Method:** All interval and screen detected cancers occurring in women recalled to assessment between 2013-2018 were reviewed. Lesion type, further views +/- biopsy, final histology and adequacy of assessments evaluated.

**Results:** Within the 5-year period 279,997 women were screened with 14,339 (5.1%) recalled for assessment. 118 were reviewed for SSA and 73 excluded due to assessment of contralateral breast or different abnormality/quadrant. The remaining 45 patients were true SSA (FNA rate 0.3%). The most common type of lesion inadequately assessed were small masses 33% (n=15) followed by micro-calcification (MCC) 22% (n=10). There was a noticeable reduction of MCC SSA from 28% to 0% in the later years due to change in practise of increasing MCC biopsies. The remaining inadequate assessments included asymmetry 4% (n=2) and distortion 2% (n=1).

**Conclusion:** SSA rates are low with improved inadequate assessments over the years within our centre. Subtle mass lesion misconstrued as benign due to false reassurance from further views and ultrasound remain most common type of SSA. Thus, audit and review of these cases are essential for education and service improvement.

### PB.33 Accuracy of breast MRI in determining the size of lobular carcinoma of the breast

#### Nuthan Devi Gupta, Mahmoud Mostafa, Jennifer-Louise Finldlay

##### Arrowe Park Hospital, Wirral, United Kingdom

###### **Correspondence:** Nuthan Devi Gupta

**Background:** Breast MRI is a useful adjunct to mammography and ultrasound in diagnosis and management of lobular breast cancer (LBC).

**Aims and objectives:** To compare the size of LBC on breast MRI and histopathology.

**Methods:** MRI and histopathological data were collected for patients from core biopsy register over 3 years from January 2015 to December 2017. Concordance ratio (CR) was calculated as tumour size on MRI / histopathological size. Ideally CR should be 1. We expect most of calculated CR to fall within 1 standard deviation (SD) i.e. 0.65 to 1.34.

**Results:** Out of the 136 patients with biopsy proven LBC, MRI and histopathological data was available in 66 patients. Median age was 62 years (range 41 to 87). Of the 66 patients, 36 had MRI for assessment, 27 for symptomatic and 3 for surveillance purposes. CR was not calculated in 9 patients due to either the size being not available or because it was diffuse/ multifocal. In 57 patients, median CR was 0.92, mean 0.9 and range 0.1 to 1.93. 36 of these were within 1 SD and another 21 patients were outside of it.

**Conclusion:** In our cohort, it was possible to calculate CR in 57 out of 66 patients. We felt that in 54% of patients, the size of the tumour on MRI was comparable to the histopathological size.

Breast MRI plays a key role in preoperative assessment of tumour size, in detecting contralateral breast cancer and to determine if tumour is multifocal/ multi centric.

### PB.34 Tumour characteristics of patients with CT-detected internal mammary lymph node and sternal metastatic disease from breast cancer

#### Reena Aggarwal^1,2^, Joanne York^1^, Anne Turnbull^1^

##### ^1^Royal Derby Hospital, Derby, United Kingdom. ^2^University Hospitals of Leicester, Leicester, United Kingdom

###### **Correspondence:** Reena Aggarwal

**Background:** Internal mammary lymph node (IMN) metastases have a similar prognostic significance to axillary nodal metastases in breast cancer. IMN radiotherpy has a potential to reduce recurrence and improve survival, however there is an increased risk of toxicity. It is therefore important to identify patients at higher risk of IMN metastases.

**Method:** A retrospective search for all patients with first presentation of metastatic breast cancer from 2012 to 2017, was performed using our local metastatic breast cancer MDT database (n=229). Staging CT images were reviewed for IMN and/or sternal soft tissue disease. Histology was reviewed and information including location of primary tumour, grade, receptor status and axillary nodal involvement was collected.

**Results:** 47 patients (20.7%) had involvement of IMNs/sternal soft tissue, however histology for 10 was not available. 37 patients were included in this study.

20 (54.1%) primary tumours were in the outer breast, 12 (32.4%) in the inner breast. 26 (70.3%) of tumours were grade 3, 8 (21.6%) grade 2 and 3 (8.1%) grade 1. Majority of tumours were HER2 negative (n=31, 83.8%), with no significant difference in ER status. 12 (32.4%) were triple negative. 10 (27%) patients had >= 4, 14 (37.8%) had <4 and 13 (35.1%) had 0 axillary metastases. Most patients had a moderate (35.1%) or poor (48.6%) Nottingham Prognostic Index.

**Conclusion:** Grade 3, HER 2 negative/triple negative disease is most commonly associated with IMN/sternal soft tissue metastases. It is important to identify these patients so that local radiotherapy can be considered.

### PB.35 The role of magnetic resonance imaging in preoperative planning for patients with multifocal or multicentric breast cancer

#### Stacey Jones, Margaret Fletcher, Nisha Sharma

##### St James's University Hospital, Leeds, United Kingdom

###### **Correspondence:** Nisha Sharma

**Introduction:** Traditionally based on clinical assessment and conventional imaging, role of breast magnetic resonance imaging (MRI) in multicentric breast cancer (MCBC) and multifocal breast cancer (MFBC) is not fully established.

**Aims:** Assess role of MRI in preoperative planning of women with MFBC and MCBC who are potentially suitable for breast conservation surgery (BCS) and survey Breast Surgeons on management of MFBC and MCBC.

**Methods:** 63 women with MCBC and MFBC diagnosed between March 2014 and 2015 were identified. Patients suitable for BCS were assessed at MDT with descision for preoperative MRI made. Contribution of MRI to disease assessment and surgical outcome was reviewed. Questionnaire survey was given to all Breast Surgeons in the unit to determine preferred management of conservable MCBC and MFBC.

**Results:** 23/63 women had preoperative MRI in addition to conventional imaging. 40 ladies who did not have an MRI, 70% underwent a mastectomy and 5% underwent BCS. 23 women that had MRI, 48% had mastectomy and 30% had BCS. BCS was performed in all patients suggested by MRI. 86% of surgeons would perform BCS on individuals with MFBC, in comparison to 29% in MCBC.

**Conclusion:** MRI correlates more closely with histological size than conventional imaging, allowing for correct surgical outcome. MRI should be used to aid surgical planning in individuals wishing to have two separate WLEs and avoiding overtreating with mastectomy. Majority of Surgeons are reluctant to perform BCS in potentially conservable MCBC. Therefore, a cultural change amongst surgeons to support BCS in this context is needed.

### PB.36 Improving accuracy of ultrasound-guided needle localisation of breast lesions – does the type of needle make a difference?

#### James Russell, Elli Papantoniou, Glenda Kaplan

##### Royal Free London NHS Foundation Trust, London, United Kingdom

###### **Correspondence:** Elli Papantoniou

**Background:** Many breast lesions initially detected by mammography are small and subsequently impalpable. These lesions must be accurately localised pre-operatively using a needle and guidewire to ensure successful excision and conservation of normal breast tissue. At our institution, ultrasound is the preferred modality for needle localisation, with the type of needle used largely based on individual surgeon preference.

**Aim:** Our aim was to ascertain which factors influence accuracy of ultrasound-guided needle localisation, specifically whether there is a correlation between type of needle used and accuracy of placement.

**Methods:** A total of 100 ultrasound-guided needle localisations performed by two different consultant radiologists were included. For each case, we documented the type of needle used, the distance of the needle tip from the lesion, the nature of the lesion, and whether or not the lesion was successfully excised.

**Results:** Two types of needle were used; the Homer Mammalok needle (n=80), and the Hawkins II needle (n=20). Using the Homer Mammalok needle, the mean distance of the needle tip from the lesion was less than 10mm in 94% of cases (mean=6mm). Using the Hawkins II needle, the mean distance of the needle tip from the lesion was less than 10mm in 35% of cases (mean=12.9mm). No other factors were found to significantly influence accuracy of localisation.

**Conclusion:** Our results suggest that the Homer Mammalok needle provides more accurate localisation than the Hawkins II needle. Whilst using the Homer Mammalok needle alone would improve accuracy of localisation, individual surgeon preference remains an important factor.

### PB.37 The impact of axillary lymph node cortical thickness on predicting axillary nodal metastasis in breast cancer

#### Stacey Jones, Nisha Sharma

##### St James's University Hospital, Leeds, United Kingdom

###### **Correspondence:** Nisha Sharma

**Introduction:** Axillary lymph node (ALN) status is an important prognostic factor in breast cancer and crucial in treatment decisions. Individuals without suspicious ALNs undergo sentinel lymph node biopsy (SLNB) whereas, positive ALNs proceed to axillary lymph node clearance (ALNC).

**Aims:** To determine ALN cortical thickness cut-off that is predictive of nodal disease and if number of abnormal ALNs on axillary ultrasound (AUS) is predictive of number of positive ALNs at histology, in order to identify patients with low volume disease who may be offered SLNB over ALNC.

**Methods:** Between October 2017 and May 2018 preoperative AUS was performed in 217 patients scheduled for surgery. Cortical thickness and number of nodes established via AUS and compared with histology.

**Results:**

217 AUS performed, 71% normal, 21% equivocal and 8% had an abnormal AUS. 19% of normal AUS had false negative result. 63/217 underwent nodal FNA. 23/63 had nodal metastases whereas 40/63 did not. However, 7/40 had false negative FNA results compared to histology.

ALN cortical thickness cut-off of 2mm, 63 ladies underwent FNA with 23 proceeding with ANC. Increasing cut-off to 4mm, 20 ALNs underwent FNA with 14 ALNCs. Lastly, 5mm cortical thickness cut-off, 16 ALN FNAs performed and 13 ALNCs.

14/23 had one abnormal ALN while 9/23 had multiple abnormal ALNs on AUS. 74% had ≤4 metastatic ALNs at histology after ALNC.

**Conclusion:** Increasing cortical thickness threshold from 2mm to 5mm, we would reduce number of patients undergoing unnecessary FNAs and avoid overtreating by upfront ANC thereby reducing incidence of lymphoedema.

### PB.38 Audit of B3 lesions in a breast symptomatic service

#### Anwen Newland^1^, Lubna Khalid^1^, Angela Gupta^2^, Sylvie Flais^1^

##### ^1^London Northwest University Healthcare NHS Trust, London, United Kingdom; ^2^West of London Breast Screening Services, London, United Kingdom

###### **Correspondence:** Anwen Newland

**Introduction:** The symptomatic breast service in the UK has mostly adopted the pathway of the NHSBSP in the management of B3 lesions. The population attending the symptomatic service is different to the screening service.

**Method:** We reviewed all the B3 lesions biopsied in the symptomatic service at Ealing Hospital between January 2015 and July 2017 under US or stereotactic guidance. The histology and subsequent management were recorded.

**Results:**

64 B3 lesions were biopsied in 61 patients (11 stereotactic biopsies, 53 US guided biopsies). 22 lesions (34%) were papillomas, 20/53 (40%) in the US group, 2/11 (18%) in the stereotactic group. All lesions were managed appropriately. 5 lesions were upgraded to malignant at subsequent biopsy or surgical excision. The proportion of papilloma was compared to the proportion of papilloma found amongst B3 lesions in our local screening centre between April 2017 and March 2018: 12/111 (11 %).

**Conclusion:** The proportion of papillomas is much greater within the symptomatic than within the screening service, contrary to what has been reported previously. The malignant upgrade rate (8%) appears smaller than what is reported in the literature but this may be due to our small number of patients and the high proportion of papillomas without atypia.

### PB.39 Screening mammographic interval cancer review: how often does a second bite at the cherry taste different?

#### Dagmar Godden^1^, Harriet Russell^1^, Luci Hobson^2^, Clare Alison^1^, Barnes Debbie^1^, Helen Farmer^1^, Eleanor Cornford^1^, Iain Lyburn^1^

##### ^1^Thirlestaine Breast Centre, Cheltenham, United Kingdom; ^2^Wiltshire Breast Screening Unit, Swindon, United Kingdom

###### **Correspondence:** Dagmar Godden

**Background:** Review of interval cancers is integral in breast screening. We set out to evaluate missed features and differences between the opinions of individual readers at the time of initial screen reading compared to subsequent interval review.

**Methods:** Interval review according to PHE/NHS BSP Guidance (2017). For category 2 (seen with hindsight, difficult to perceive) and 3 (appearance of malignancy) cancers, features were recorded and opinions of readers at interval review compared with prior screening interpretation if applicable.

**Results:** 85 instances involving 9 readers fulfilled the study criteria for cancers diagnosed July 2011 - February 2018. In 53 instances (62.5%) opinions were different – return to screen at initial interpretation and recall for assessment at review; in 32 (37.5%) instances opinions were return to screen for both reads [In these cases more than one other film reader recalled the case at interval review]. Interval between screen and diagnostic mammogram: range 3 - 36 months. 76 cases were invasive cancers; 9 DCIS. Discrepancies were perceptive and interpretative. Features not recalled included density/distortion/asymmetry (67.1%), microcalcification (21.2%), rounded mass (4.7%) and other (7.0%).

**Conclusions:** Image interpretation is complex, with varying thresholds for recall between and within individuals. Temporal variability is likely to be due to a number of factors including improved experience and the context of the situation – deciphering routine screening and interval cases with a different approach for recall. Cases where the opinions were routine recall in both instances were particularly valuable – readers identified specific features where individual improvement could be made.

### PB.40 Second-Look ultrasound following MRI – what are we finding?

#### R. C. Brook, Michaela Stahnke

##### University Hospital Southampton, Southampton, United Kingdom

###### **Correspondence:** Michaela Stahnke

Breast MRI is widely used to assess extent of breast malignancy, problem solving and in screening and surveillance of high risk patients. However, MRI often generates further questions. Second-look Ultrasound (US) is a useful tool where further clarification is required.

We felt that we were generating a high proportion of further investigations following MRI and wanted to assess how we were performing against published data.  We retrospectively reviewed 12 months of second-look US from our Breast Unit, from January 2016 to December 2016. 286 MRIs performed in that time generated 83 Second-look US (29%). We describe the cohort of patients undergoing MRI, the reasons second-look US were recommended, our success at finding the corresponding abnormality, and our tissue sampling (core biopsy/FNA) rates and pathology.

Our pick up of MRI abnormalities on second-look US (76%) is in line with published data. 60% of US lead to core biopsy, of which 38% were B5 result. We discuss the impact of this on management plans in individual cases.  Our cancer detection rate on second-look US is in line with published data. We also discuss the 1-year follow-up of patients in whom no corresponding abnormality was seen on US.

**Learning objectives:**

- To recognise the limitations of Breast MRI and highlight the frequent need for subsequent investigations. Approximately a third of breast MRI required further US.
- Second-look US is a useful tool for problem solving. The detection of MRI abnormalities on US is relatively high, and where biopsied, a high proportion are malignant.

### PB.41 Audit of previously assessed cancers identified between April 2011 and March 2016 in London Screening Services

#### Tamara Suaris^1^, Mohamed Al-Sewan^2^, Nigel Barrett^3^, Elizabeth Muscat^4^, Kesthra Satchithananda^5^, Will Teh^6^, David Jeansoule^7^, Louise Wilkinson^8^

##### ^1^Central and East Breast Screening Service, London, United Kingdom; ^2^Outer and North East London Breast Screening Service, London, United Kingdom; ^3^West of London Breast Screening Service, London, United Kingdom; ^4^South West London Breast Screening Service, London, United Kingdom; ^5^South East London Breast Screening Service, London, United Kingdom; ^6^North London Breast Screening Service, London, United Kingdom; ^7^London region Screening Quality Assurance Service, London, United Kingdom; ^8^Oxford Breast Imaging Centre, Oxford, United Kingdom

###### **Correspondence:** Louise Wilkinson

**Aim of audit:** To identify cancers in region where the woman had been recalled for assessment with a benign outcome in the previous screening round, and to ascertain whether systematic review was achievable and of educational value.

**Method:** Screening directors from London region collaborated in this audit, supported by SQAS. A list of relevant interval and screen detected cancers identified within each 12 month period was sent to directors by SQAS annually, and cases were reviewed using a standard proforma. Information on mammography findings and outcome at previous assessment and at diagnosis was collected. Learning points from individual cases were recorded.

**Findings:** 475 potential cases were considered. As the cases included interval cancers notified and reviewed within the year, the screening assessments took place over an extended period from 1997 to 2015. There were 86 interval cancers, 377 cancers diagnosed at next screen and 4 cancers diagnosed at short term recall. 162 cancers were same side, same site. In 62 cases, the assessment was considered suboptimal, usually because further imaging and/or histology was interpreted as normal. Reasons for suboptimal assessment included: no interval change, no focal abnormality, benign findings at assessment, no biopsy or benign biopsy result.

Results were discussed at regional meetings and learning points disseminated to clinical teams. Changes in practice were noted over the 5 years of the audit. A recent matching exercise with cancer registry may increase the ratio of interval cancers compared to screen detected cancers.

### PB.42 The role of magnetic resonance image guided 2nd look ultrasound - effecting change in management for patients considered for breast conserving surgery

#### Andreas Panayiotou, Vandana Gaur, Muthyala Sreenivas

##### University Hospital of Coventry and Warwick, Coventry, United Kingdom

###### **Correspondence:** Andreas Panayiotou

**Introduction:** Magnetic Resonance Imaging (MRI) guided second look ultrasound (US) is an established technique for detecting areas of suspicious breast tissue adjacent to a primary breast carcinoma, distinguishing solitary from multifocal disease and detecting occult malignancy. It has the advantage of being able to identify and sample these lesions. This has a key role in determining whether breast conserving surgery or mastectomy is performed.

**Methods:** A retrospective study of 143 cases in which MRI guided 2nd look breast ultrasound was carried out from 4^th^January 2012 to 10^th^April 2018. Data was analysed for 104 cases from our institution with completed information. This included; correlation between MRI and US findings, histology results and whether patient management was impacted, in terms of solitary or multifocal disease and subsequent treatment.

**Results:** Second look US was performed in 95 cases (one case was excluded due to patient mortality). 83 out of 95 (87%) had positive 2^nd^ look US of which 43 (45%) patients had additional malignancies with subsequent change in their surgical management. US failed to identify a lesion in 22 cases and required further MRI guided biopsy - from which 4 were histologically malignant requiring a change in surgical management.

**Conclusion:** We have demonstrated that MRI guided 2nd look US is effective for the detection of incidental further tumour foci and a cost effective method of altering patient management. MRI guided biopsy has proven useful in lesions not demonstrated on 2^nd^look ultrasound.

### PB.43 NHSBSP Higher risk Screening: the experience of a UK Breast Screening programme in organising the surveillance of the higher risk population, 5 years on

#### Carol Ellen Holmes^1^, Philippa Pattison^2^, Alex Henderson^3^, Nidhi Sibal^2^

##### ^1^Newcastle Breast Screening Unit, Royal Victoria Infirmary, Newcastle upon Tyne, United Kingdom; ^2^Newcastle Breast Screening Unit, Newcastle upon Tyne, United Kingdom; ^3^Centre of Life, Genetics, Newcastle upon Tyne, United Kingdom

###### **Correspondence:** Carol Ellen Holmes

In 2007, the cancer reform strategy stated that women identified at higher risk of developing breast cancer, should be managed by the NHSBSP. This was to ensure standardisation across centres with regular intervals between screens and provide high quality screening and assessment. NHSBSP commenced the management of higher risk screening from April 2013.

The Newcastle Breast Screening programme manages the higher risk screening for its own local population and two adjacent screening programmes. Up until 2013, women who were considered at higher risk of breast cancer, in the geographic region surrounding these three screening units, were managed in the Newcastle symptomatic unit predominantly. The aim of this poster is to outline the processes involved in organising the screening of this group of women from 2013. The different steps in the process, such as risk assessment, planning and delivering high-risk screening will be discussed. Our strategies for performing mammogram and MRI in a timely manner will be outlined. Effective communication between the breast screening office, the client, genetics, the MRI department and the larger breast multidisciplinary team is crucial to ensuring a high quality service.

### PB.44 Role of vacuum assisted excision in the investigation of B3 lesions in cases where the mammographic abnormality has been removed at biopsy

#### Madhuchanda Bhattacharyya, Vaishali Parulekar

##### Oxford University Hospitals NHS Foundation Trust, Oxford, United Kingdom

###### **Correspondence:** Madhuchanda Bhattacharyya

**Introduction:** Screen-detected abnormalities visible only on mammogram, undergo 10G vacuum assisted biopsy (VAB) at our centre. B3 lesions then undergo stereotactic vacuum assisted excisions (VAE).  We reviewed B3 lesion outcome data, where the mammographic abnormality was removed at initial stereotactic VAB.

**Method:** Outcome data review of 78 B3 cases diagnosed between 2014 and 2017.

Results:

In 32 cases (41%), the radiological abnormality was removed at initial biopsy.

15/32 did not undergo VAE and are under annual 5 year follow up.  All were microcalcification (MCC) measuring <10mm.

14/32 underwent VAE. 13/14 cases were microcalcification (MCC) and 1/14 was a mass with MCC. The mammographic abnormality was <10mm in 13/14 cases and 1/14 was 14mm. Following VAE, atypia remained unchanged from original biopsy in 4 /13 cases. 10/14 showed benign changes only. Atypia was incidental in 5 cases but benign on VAE. In 9 cases, atypia represented the radiological abnormality and in 4/9 further excision showed the same findings. Follow up between 1-3 years has resulted in one cancer detected at year 3 in the same breast but at a different site.

No upgrades were identified at VAE for any cases where the radiological abnormality was removed at VAB.

3 underwent surgical excision of which 2 showed benign changes only. One was excised along with co-existent DCIS

**Conclusion:** Our study found that once the mammographic abnormality was removed by VAB, subsequent VAE did not lead to upgrade, even where atypia was incidental to radiological abnormality. Most cases showed benign changes only.

### PB.45 Upgrade rates of B3 and B5a diagnoses on diagnostic needle core and vacuum assisted biopsies at our unit

#### Seema Salehi-Bird, Feyi Babatola, James Davies, Rachael Gelder, Zatinahhayu Mohd-Isa

##### University Hospitals North Midlands, Stoke-on-Trent, United Kingdom

###### **Correspondence:** Rachael Gelder

The number of vacuum assisted biopsies performed in our unit continues to increase. Anecdotally there is an increasing B3 diagnosis rate on both needle core biopsy (NCB) and first line vacuum assisted biopsy (VAB). According to NHSBSP guidelines for the diagnosis and management of lesions of uncertain malignant potential on core biopsy, vacuum assisted excision (VAE) should be performed to ensure a B3 lesion has been thoroughly sampled. The NHSBSP and Association of Breast Surgery audit of screen detected breast cancers demonstrated an upgrade rate of 12% from non-invasive (B5a) on diagnostic core biopsy to invasive cancer at surgery for the screening year April 2016 to March 2017.

We wished to retrospectively review our vacuum assisted biopsies including the factors above over the last 3 years including both symptomatic and screening women.

398 patients were biopsied over the 3 year period 1/7/2015 to 30/6/2018 inclusive. Total number of needle core biopsies (NCB) = 226 and total number of vacuum procedures = 424.

16 NCBs were diagnosed as B5a, 1 (6%) was upgraded to invasive disease at surgery. 41 VABs were diagnosed as B5a of which 2 (4%) were upgraded at surgery. 33 VAEs were diagnosed as B5a of which 1 (3%) was upgraded at surgery. Analysis of B3 data demonstrates a 14% (25/153) upgrade rate from B3 on NCB and a 7% (4/55) upgrade rate from B3 on VAB to either suspicious, in-situ or invasive cancer at VAE, confirmed at surgery.

Our results are within national guidelines.

### PB.46 Withdrawn

### PB.47 The accuracy of axillary nodal ultrasound and ultrasound fna/core biopsy in the pre-operative staging of patients with lobular breast cancer- re-audit of our experience

#### Theodora Zachari, Gauri Babu

##### Western General Hospital, Edinburgh, United Kingdom

###### **Correspondence:** Theodora Zachari

The purpose of this retrospective study was to determine the accuracy of preoperative axillary sonography in patients with invasive lobular cancer (ILC)

Patients who underwent surgery for ILC from January 2014 to December 2016 were identified from postoperative histological records.

We identified 253 cases of lobular cancers of which 71 patient were node positive.

Of these 50 % percent of node positive cases had an abnormal axillary USS. Two thirds had a core biopsy to prove nodal involvement (i.e. 20 patients). However, 9 of this had a conversion from FNA to core biopsy.

Axillary disease in lobular cancers may differ slightly from NST type. The disease is sometimes difficult to quantify on standard imaging. However, we have to underline that the core biopsy is more accurate in the diagnosis of axillary nodal disease.

In ILC, metastases are thought to be difficult to detect because the cells are small and on cytology resembles lymphocytes.

In the above data collection, we found that our accuracy rate for lobular cancers was slightly variable from NST. We are in the process of reviewing our practice to ascertain if we need to proceed to core biopsy instead of FNA in proven lobular malignancies and revisit the axilla after pathology available.

### PB.48 Can the upper age limit for avoiding biopsies in benign breast lesions be safely increased to 30 years of age from the current threshold of 25 years?

#### Kirsty MacGregor^1^, Muthyala Sreenivas^2^

##### ^1^Warwick Medical School, Coventry, United Kingdom; ^2^UHCW NHS Trust, Coventry, United Kingdom

###### **Correspondence:** Kirsty MacGregor

**Introduction:** In this single institute retrospective study we aimed to determine whether not performing a biopsy in women up to 30 years from the current standard of up to 25 years was safe when Stavros criteria are strictly applied.

**Methods:** 317 lesions which were biopsied under US guidance over 7 years in women between the ages of 25 and 30 years. A separate prospective review was performed on the stored US images using Stavros criteria. Results from this review were compared with original radiological assessment.

**Results:** There were 15 malignant lesions on prospective review and all of these violated Stavros criteria for a benign lesion. 228/302 histologically benign lesions fulfilled Stavros criteria for benignity whilst the remainder violated benignity criteria. The sensitivity of the criteria to identify lesions as benign was 75% and specificity was 100%. Original and review radiological grading for each lesion showed that there were 243, 67 and 7 R2, R3 and R4/5 categories in the original grading versus 224, 71 and 22 such respective categories in the reviewed grading with many cancers occurring in the revised R4/5 category.

**Conclusion:** This study demonstrates that when Stavros criteria are strictly adhered to, the safe age threshold for not performing a biopsy in radiologically benign lesions can be increased to 30 years. At our institute, this equates to a cost saving of approximately £77,748 over the 7 year study period.

### PB.49 Withdrawn

### PB.50 Fat necrosis in the breast with no history of trauma: do we need to biopsy?

#### Jones Matthew, Nest Evans

##### Aneurin Bevan Health Board, Newport, United Kingdom

###### **Correspondence:** Nest Evans

Our policy at ABUHB has been to biopsy probable fat necrosis if there is no history of trauma or surgery. This is a relatively common presentation in our symptomatic service.

**Audit Purpose:** In the presence of typical ultrasound changes of fat necrosis (defined and illustrated in the poster) without antecedent - is a biopsy necessary?

**Method:** 2 year retrospective review of ALL breast ultrasound reports containing the words fat necrosis.

**Results:** After some initial exclusions -a total of 240 Cases were reviewed (population covered is 650,000) FINDINGS: 73 biopsies taken. Benign results obtained in 70 A conclusive diagnosis of fat necrosis on US had a 85% PPV for fat necrosis on subsequent and 100% PPV for benign disease. In the 3 cases of malignancy detected - the ultrasound report included fat necrosis in the differential but there was significant concern re other pathology and biopsy would always be performed. 167 cases were not biopsied and 73% of these had a clear history of trauma .There were however 27% of cases who had no history of trauma - some of whom were managed with clinical and US follow up .No other pathology or adverse events were seen in this group.

**Conclusion:** Criteria for a positive diagnosis of fat necrosis based on ultrasound appearance will be outlined. Our study suggests that classical fat necrosis in the absence of trauma or surgery does not need to be biopsied as US change has a high PPV value for fat necrosis and 100%PPV for benign disease.

